# Comprehensive genomic and functional characterization of a phytoplasma associated with root retardation, early bolting, witches’-broom, and phyllody in daikon (*Raphanus sativus* L.)

**DOI:** 10.3389/fmicb.2025.1654928

**Published:** 2025-09-09

**Authors:** Yi-Ching Chiu, Yu-Chen Lin, Shang-Hung Pao, Yuh-Kun Chen, Pei-Qing Liao, Helen Mae Mejia, Chiou-Rong Sheue, Chih-Horng Kuo, Jun-Yi Yang

**Affiliations:** ^1^Doctoral Program in Microbial Genomics, National Chung Hsing University and Academia Sinica, Taichung, Taiwan; ^2^Institute of Plant and Microbial Biology, Academia Sinica, Taipei, Taiwan; ^3^Department of Life Science, National Chung Hsing University, Taichung, Taiwan; ^4^Department of Plant Pathology, National Chung Hsing University, Taichung, Taiwan; ^5^Institute of Biochemistry, National Chung Hsing University, Taichung, Taiwan; ^6^Institute of Biotechnology, National Chung Hsing University, Taichung, Taiwan; ^7^Advanced Plant and Food Crop Biotechnology Center, National Chung Hsing University, Taichung, Taiwan

**Keywords:** phytoplasma, daikon, PHYL1, SAP11, auxin

## Abstract

Daikon (*Raphanus sativus* L. var. *longipinnatus*) is an economically important root crop and medicinal plant. In 2021, a previously unreported disease outbreak characterized by early bolting, witches’-broom, phyllody, virescence, and severe root growth retardation was observed in daikon fields in Yunlin, Taiwan. Transmission electron microscopy revealed pleomorphic phytoplasma-like bodies within the sieve elements of symptomatic plants. Molecular diagnostics and phylogenetic analyses identified the causal agent as a 16SrII-A subgroup strain of ‘*Candidatus* Phytoplasma aurantifolia’, designated NCHU2022. Hybrid genome assembly using Illumina and Oxford Nanopore sequencing yielded a complete genome consisting of a 632 kb circular chromosome and a 4.2 kb plasmid. Effector prediction and functional assays identified two secreted proteins, SRP06 and SRP15, homologous to SAP54/PHYL1 and SAP11, respectively, which induced phyllody and witches’-broom symptoms through destabilization of floral MADS-domain and class II TCP transcription factors. Transcriptomic profiling of infected roots revealed activation of auxin biosynthesis and signaling pathways, accompanied by suppression of cytokinin signaling and induction of lignin biosynthesis, suggesting that hormonal reprogramming contributes to abnormal root development. While previous studies have primarily focused on aerial symptomatology—such as witches’-broom, phyllody, and sterility—our findings highlight an underexplored dimension of phytoplasma pathology: the profound impairment of root development driven by auxin dysregulation and associated transcriptional remodeling.

## Introduction

Phytoplasmas are pleomorphic, phloem-restricted bacterial pathogens transmitted by phloem-feeding insects, such as leafhoppers, planthoppers, and psyllids (Trivellone and Dietrich, 2021). To date, 44 phytoplasma species have been classified in the genus ‘*Candidatus* Phytoplasma’ based primarily on the similarity of the 16S rRNA gene (Namba, 2019). Alternatively, they can be grouped using *i*PhyClassifier, a virtual restriction fragment length polymorphism (RFLP) profiling tool that aids in subgroup designation (Zhao et al., 2009). These bacteria infect over 700 plant species and are responsible for a range of disease symptoms, including stunting, yellowing, virescence, phyllody, witches’-broom, vivipary, purple top, and general decline (Kumari et al., 2019; Namba, 2019). In Taiwan, phytoplasmas are known to infect a wide range of plant species, including vegetables, ornamentals, medicinal herbs, and weeds, with identified strains belonging to groups such as 16SrI, 16SrII, 16SrVIII, 16SrX, 16SrXI, 16SrXII, and 16SrXIV (Yang et al., 2023).

The virulence factors secreted by phytoplasmas, SAP11, SAP54/PHYL1, TENGU, and SAP05, have been known to reprogram host development and defense by targeting key transcription factors and hormone signaling pathways (Sugio et al., 2011b; Wang R. et al., 2024). Among them, SAP54/PHYL1 induces phyllody by promoting proteasomal degradation of floral MADS-box transcription factors (MTFs) through RAD23-mediated recruitment (MacLean et al., 2014; Maejima et al., 2014). SAP05 prolongs the vegetative phase by facilitating ubiquitin-independent degradation of SQUAMOSA promoter-binding protein-like (SPL) transcription factors (Huang et al., 2021). SAP11 destabilizes class II TCP (TEOSINTE BRANCHED1, CYCLOIDEA, and PROLIFERATING CELL FACTOR 1 and 2) transcription factors, thereby promoting axillary meristem proliferation and suppressing jasmonic acid (JA) biosynthesis through downregulation of *LOX2*, a JA biosynthetic gene (Chang et al., 2018; Sugio et al., 2011a). TENGU, predominantly found in 16SrI phytoplasma strains, induces floral sterility and dwarfism by repressing auxin signaling via inhibition of *ARF6* and *ARF8* expression (Hoshi et al., 2009; Minato et al., 2014).

Daikon (*Raphanus sativus* L. var. *longipinnatus*) is a widely cultivated root vegetable belonging to the Brassicaceae family, appreciated not only for culinary applications but also for its medicinal properties in traditional Asian medicine (Baenas et al., 2016; Shukla et al., 2011). The formation of storage roots is a precisely coordinated developmental process, encompassing cell division, expansion, differentiation, and biomass accumulation. Among phytohormones, auxin is particularly critical, playing a central regulatory role across these developmental stages (Kondhare et al., 2021). Recent research has further connected auxin signaling to lignin biosynthesis, highlighting how hormonal regulation influences cell wall composition and tissue rigidity (Wang W. et al., 2024). Thus, maintaining finely-tuned auxin homeostasis is essential for proper root morphogenesis.

In Taiwan, daikon cultivation is threatened by various pathogens, including *Xanthomonas campestris* pv. *campestris* (black rot), *Erwinia carotovora* (soft rot), *Fusarium oxysporum* f. sp. *raphani* (yellows), and radish mosaic virus (Chuang et al., 1989; Lo and Sun, 1987; Wang et al., 2014). In this study, the first genomic and functional characterization of a phytoplasma associated with witches’-broom disease in daikon was presented. Using a combination of genome sequencing, effector characterization, and transcriptomic profiling, the molecular mechanisms underlying disease symptom development and host responses were examined. Our findings provide a comprehensive view of how phytoplasma infection can induce host developmental reprogramming through key virulence factors and hormone signaling disruption, with unique manifestations in root-dominant crops like daikon.

## Materials and methods

### Sampling

Early bolting and witches’-broom symptoms were observed in three commercial daikon cultivation areas in Mailiao Township, Yunlin County, Taiwan: Site I (23°45′44.7″N, 120°15′00.1″E), Site II (23°45′56.2″N, 120°14′49.4″E), and Site III (23°45′13.0″N, 120°15′28.2″E). The disease incidence was estimated at 0.56% (88 symptomatic plants out of 15,465), 0.25% (22/8,774), and 0.16% (117/73,125) at Sites I, II, and III, respectively. A total of 11 symptomatic plants were selected for molecular analyses, including 4 from Site I, 3 from Site II, and 4 from Site III, and used for DNA extraction and sequencing. To serve as healthy controls, five asymptomatic daikon plants were collected from the same three sites during the same period, including 2 from Site I, 1 from Site II, and 3 from Site III, ensuring that all control samples were matched for growth stage, soil type, and environmental conditions.

### Polymerase chain reaction (PCR) and nested PCR

The genomic DNA (gDNA) from the leaves of healthy and symptomatic plants was extracted by the Plant Genomic DNA Purification Kit following the manufacturer’s instructions (DP022-150, GeneMark). The gDNA was examined by nested PCR using the phytoplasma universal primer pairs P1/P7 followed by R16F2n/R16R2 to amplify a 1.2 kb DNA fragment of the 16S ribosomal RNA (rRNA) gene (Lee et al., 1993). The first round of nested PCR was carried out for 12 cycles in a final volume of 20 μL, in which 1 μL of the product was used as a template for the second round PCR executed for 35 cycles. The P1/P7 primer pair-amplified DNA fragments were further sequenced with P1 and a nested primer. To investigate the genetic correlation of the 16SrII-A subgroup phytoplasma strains associated with different plant diseases found in Yunlin, specific primer sets designed according to the genome information of the ‘*Ca.* P. aurantifolia’ NCHU2014 (accession No. CP040925) and ‘*Ca.* P. aurantifolia’ NTU2011 (accession No. NZ_AMWZ01000001.1–13.1) were used for PCR analyses. The primer sequences are listed in Supplementary Table S1.

### Transmission electron microscopy

The procedure for transmission electron microscopy (TEM) was conducted as described previously with modification (Buxa et al., 2015). In brief, symptomatic samples were fixed with 2.5% glutaraldehyde in 0.1 M phosphate buffer (pH 7.2) and post-fixed with 1% osmium tetraoxide. Samples were then dehydrated with an ethanol series and immersed in LR White Resin. Ultrathin sections were cut using an ultramicrotome and stained with uranyl acetate and lead citrate. Then, sections were observed under a JEOL JEM-1400 series 120 kV Transmission Electron Microscope (Jeol), and photos were collected by a Gatan Orius SC 1000B bottom mounted CCD-camera (Gatan Inc.).

### Western blotting

Samples were collected and ground in liquid nitrogen. Total cell extracts were prepared by directly adding 2.5 × SDS sample buffer (5 mM EDTA, 5% SDS, 0.3 M Tris–HCl, pH 6.8, 20% glycerol, 1% β-mercaptoethanol, and bromophenyl blue) into ground samples, which were heated at 95°C in a dry bath for 10 min. After centrifugation at 13,000×*g* for 12 min, supernatants were obtained as total cell extracts, and proteins were separated by SDS-PAGE. Polyclonal antibodies against Imp and PHYL1 were used to monitor protein amounts (Tan et al., 2021; Liao et al., 2022). Western blotting was performed using enhanced chemiluminescence western-blotting reagents (Amersham), and chemiluminescence signals were captured using ImageQuant LAS 4000 Mini (GE Healthcare).

### Phylogenetic tree construction

Phylogenetic trees were reconstructed using MEGA-X software based on the sequence comparisons of 16S rRNA gene, PHYL1 homologs, or SAP11 homologs from different phytoplasma species. Multiple sequence alignments were performed using the ClustalW program and then processed to generate neighbor-joining phylogenies with bootstrapping to perform molecular evolutionary analysis. The numbers at the branch points are bootstrap values representing the percentages of replicate trees based on 1,000 repeats.

### *i*PhyClassifier analysis

Virtual RFLP patterns was generated by *in silico* digestion of the 1.2 kb DNA fragment (R16F2n/R16R2) of the 16S rRNA gene identified from *Raphanus sativus* L. witches’-broom disease phytoplasma (accession No. OK491387) using *i*PhyClassifier, an interactive online tool^1^ (Zhao et al., 2009).

### Oxford Nanopore sequencing and Illumina sequencing

Genomic DNA was extracted from aerial tissues, including shoots, leaves, and flowers, of symptomatic *Raphanus sativus* plants. Oxford Nanopore sequencing was performed using four MinION flow cells (Oxford Nanopore Technologies, UK), with libraries prepared according to the manufacturer’s protocol (SQK-LSK109). To optimize read length, four libraries (A1–A4) were constructed using different size-selection strategies. Library A1 was sequenced without size selection. For library A2, DNA fragments smaller than 1 kb were removed using KAPA HyperPure Beads (Roche). For libraries A3 and A4, fragments below 10 kb were depleted using Short Read Eliminator (SRE) and SRE XL kits (Circulomics, USA). In parallel, Illumina paired-end libraries (150 bp) were prepared using the NEBNext Ultra DNA Library Prep Kit (New England Biolabs, USA) and sequenced on the Illumina HiSeq 4000 platform. The resulting short reads were used for hybrid genome assembly and error correction.

### Genome assembly

To initiate genome assembly, raw Illumina and Oxford Nanopore Technologies (ONT) reads were first filtered for phytoplasma-origin sequences using ‘*Ca.* P. aurantifolia’ NCHU2014 as a reference. Illumina reads were aligned using BWA v0.7.17 (Li and Durbin, 2009) with an alignment score cutoff of 30, while ONT reads were aligned using Minimap2 v2.15 (Li, 2018) with an alignment score cutoff of 1,000. All mapped reads were aligned to the NCHU2014 reference genome, and the mapping results were evaluated programmatically using the “mpileup” function in SAMtools v1.9 (Li et al., 2009), and manually inspected using IGV v2.5.0 (Robinson et al., 2011) to identify potential assembly errors. Regions with inconsistencies were manually split and rearranged based on the continuity of ONT long reads, followed by iterative validation using new mapping results. In the early assembly stages, ONT reads provided scaffolding information and supported the overall chromosomal architecture, particularly at junctions between repetitive and unique regions. Reads mapped to contig termini were visually examined in IGV to manually select representative long reads for contig extension and gap closure. In later iterations, Illumina reads were used to correct base-level errors and validate small indels introduced during long-read sequencing. This process was repeated until the final circular chromosome and plasmid assembly was confirmed, with full read support across all genomic regions. Sequencing depth was calculated using the “depth” function in SAMtools. To estimate genome size based on k-mer distribution, all Illumina reads that mapped to the final assembly with an alignment score >200 were extracted. K-mer frequencies (*k* = 17–63) were computed using Jellyfish v2.2.8 (Marçais and Kingsford, 2011), and the genome size was estimated by dividing the total k-mer count by the peak depth as described by Lu et al. (2016).

### Genome annotation

Gene prediction was carried out using RNAmmer v1.2 for rRNAs (Lagesen et al., 2007), tRNAscan-SE v1.3.1 for tRNAs (Lowe and Eddy, 1997), and Prodigal v2.6.3 for coding sequences (Hyatt et al., 2010). Gene annotation was based on homologous clusters identified from other phytoplasma genomes using BLASTP v2.10.0 (Camacho et al., 2009) and OrthoMCL v1.3 (Li et al., 2003), followed by manual curation using public databases including GenBank (Benson et al., 2018), KEGG (Kanehisa et al., 2010), and COG (Tatusov et al., 2003). Putative secreted proteins were predicted using SignalP v6.0 (Armenteros et al., 2019) under the Gram-positive bacteria model. Proteins with predicted transmembrane domains (as identified by TMHMM v2.0; Krogh et al., 2001) were excluded. Remaining candidates were further filtered to retain only those with a signal peptide length between 21 and 52 amino acids. The genome map was visualized using Circos v0.69-6 (Krzywinski et al., 2009).

### Quantitative real-time PCR (qRT-PCR) and statistical analysis

Total RNA was extracted using TRIzol™ reagent (Invitrogen) according to the manufacturer’s instructions. RNA samples were treated with DNase I (Takara, Japan) at 37°C for 30 min to eliminate genomic DNA contamination. First-strand cDNA was synthesized from 1 μg of total RNA using the SuperScript™ III First-Strand Synthesis SuperMix (Invitrogen) with a combination of random hexamers and oligo(dT)₍₂₀₎ primers. Reverse transcription was carried out at 25°C for 10 min, followed by 50°C for 40 min. Quantitative real-time PCR was performed using the KAPA SYBR® Fast qPCR Kit (Kapa Biosystems) on a CFX96™ Real-Time PCR Detection System (Bio-Rad). The thermal cycling conditions were: 95°C for 3 min (initial denaturation), followed by 40 cycles of 95°C for 10 s and 55°C for 30 s. Each reaction was conducted in three technical replicates. Samples were obtained from three biologically independent healthy and symptomatic radish plants, with one plant representing each biological replicate. Relative gene expression levels were calculated using the 2^−ΔΔCt^ method and normalized to the expression of the reference gene *Actin*. Statistical significance between healthy and diseased samples was assessed using a two-tailed Student’s t-test. Primer sequences used for qRT-PCR are listed in Supplementary Table S1.

### RNA sequencing and transcriptomic analysis

**Figure 1:**
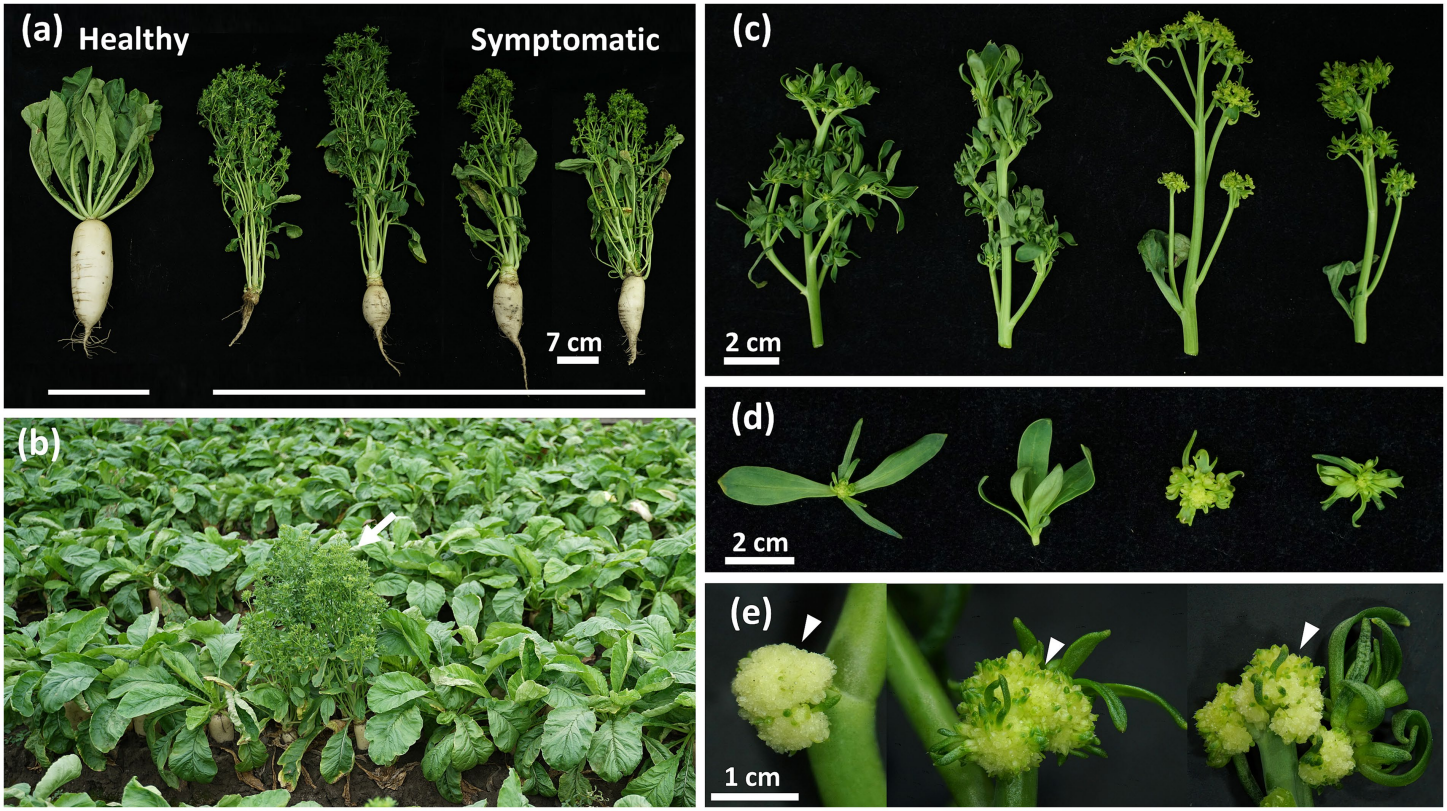
Phytoplasma-induced disease symptoms in daikon (*Raphanus sativus* L.). **(a)** Arrow indicates the symptomatic daikon with early-bolting symptom found in the field (23°45′13.0″N, 120°15′28.2″E). **(b)** A significant reduction in the root development of symptomatic daikon compared to the healthy one (left). **(c)** Phyllody and virescence symptoms with different severity observed in symptomatic daikon. **(d)** Close-up view of green leaf-like flowers in symptomatic daikon. **(e)** Arrowheads indicate the undeveloped flower buds with a cauliflower-like appearance in symptomatic daikon.

For transcriptome analysis, root tissues were collected from mature daikon plants, including healthy roots and symptomatic roots of RsWB-infected plants (Figure 1a), from Site I (23°45′44.7″N, 120°15′00.1″E). To capture the overall root response, each biological replicate was prepared by pooling tissues from the upper, middle, and lower segments of a single storage root. Three independent biological replicates were obtained for both healthy and symptomatic plants (*n* = 3 per condition). Total RNA was extracted using the RNeasy Plant Mini Kit (Qiagen) according to the manufacturer’s protocol. RNA quality was assessed by 1.2% (w/v) formaldehyde-agarose gel electrophoresis and using the Experion RNA analysis system (Bio-Rad, Munich). Only high-quality RNA samples were used for library construction and sequencing. Next-generation sequencing was performed on the Illumina HiSeq 4000 platform using 150 bp paired-end reads. For each dataset (Healthy and infection), approximately 100 million reads were generated. *De novo* transcriptome assembly was conducted, and the resulting transcripts were annotated by BlastX searches against the UniProt database.

Gene expression levels were normalized as fragments per kilobase of transcript per million mapped reads (FPKM). Differentially expressed genes (DEGs) were identified using a false discovery rate (FDR) threshold of <0.05 and a log_2_ fold change (log_2_FC) > 1 or <−1 (Supplementary Tables S2, S3).

## Results

### Identification and classification of the phytoplasma associated with the diseased daikon

In the summer of 2021, commercial cultivation of a local daikon cultivar named “YONG SIANG” was damaged by a disease exhibiting an obvious phenotype with severe root growth retardation, which was previously undocumented in Taiwan (Figure 1a). The diseased plants also displayed symptoms with early-bolting, witches’ broom, phyllody, virescence, and shoot proliferation from floral organs (Figures 1b–d). In addition, plants with severe symptoms further produced cauliflower-like heads with undeveloped flower buds (Figure 1e). These morphological changes such as witches’ broom, phyllody, and virescence displayed the typical symptoms observed in phytoplasma diseases.

The diseased daikon was observed in three planting areas of Mailiao, Yunlin County with 0.56% (88 out of 15,465), 0.25% (22 out of 8,774), and 0.16% (117 out of 73,125) incidence rate, respectively. To confirm the presence of phytoplasma, symptomatic leaves of the diseased daikon were collected and examined under the transmission electron microscope. Indeed, the pleomorphic phytoplasma of about 200–800 nm in size was found in the sieve elements (Supplementary Figure S1). Further examination of the symptomatic daikon by nested PCR and western blotting revealed that the specific phytoplasma 16S rRNA gene and immunodominant membrane protein (Imp) were only observed in the symptomatic plants (Supplementary Figures S2a,b). DNA fragments of 16S rRNA gene obtained from symptomatic daikon of a total of 11 plants in three different planting areas were sequenced and shared 100% sequence identity to each other. The DNA sequence was then deposited to Genbank under accession No. OK491387 as a partial sequence of 16S rRNA gene of *R. sativus* L. witches’ broom (RsWB) phytoplasma. Further analysis by *i*PhyClassifier revealed that the virtual RFLP pattern of the 16S rRNA sequence of RsWB phytoplasma can be classified into the 16SrII-A subgroup (Supplementary Figure S3). To illustrate the evolutionary relationship between RsWB phytoplasma and the existing phytoplasmas identified in Taiwan, a phylogenetic tree based on the sequence comparison of phytoplasmas’ 16S rRNA gene was generated. As shown in Supplementary Figure S2c, RsWB phytoplasma displayed a high sequence identity to other strains within the 16SrII group phytoplasma identified in Taiwan.

### Genetic correlation of the phytoplasmas found in different host species in Mailiao, Taiwan

Aside from daikon, economically important crops and weeds, such as peanut, mungbean, soybean, *Ixeris chinensis*, *Desmodium triflorum*, *Emilia sonchifolia*, *Nicotiana plumbaginifolia* Viv., *Digera muricata* L., *Parthenium hysterophorus* L., *Scaevola taccada*, *Celosia argentea* L., and *Eclipta prostrata*, were found to be infected by the 16SrII group phytoplasmas in or near Mailiao area (Chen et al., 2021; Chien et al., 2021a,b,c; Liao et al., 2022; Liu et al., 2015; Mejia et al., 2022; Wang et al., 2021). To investigate the genetic correlation of the 16SrII group phytoplasmas associated with different plant diseases (Supplementary Figure S4a), molecular markers, in addition to the 16S rRNA gene, for the classification of phytoplasmas were examined by PCR and DNA sequencing (Cho et al., 2020). DNA fragments of *Imp*, *tufB*, *rluA*, *degV*, *dnaD*, and TIGR00282 were amplified only from the symptomatic plants (#1 to #13) infected by the 16SrII group phytoplasmas collected from Mailiao, but not from the symptomatic loofah (#14) infected by the 16SrVIII group ‘*Ca.* P. luffae’ NCHU2019 collected from Dacheng as well as from healthy daikon (Supplementary Figures S4b–g) (Huang et al., 2022). As a control, the 16S rRNA gene were amplified from all symptomatic plants (#1 to #14) using the phytoplasma universal primer pair (Supplementary Figure S4h). The PCR amplicons were further sequenced. Comparative nucleotide sequence analyses revealed that the molecular markers amplified from the 16SrII group phytoplasma-infected plants (#1 to #13) shared 100% sequence identity (Supplementary Table S4). These results suggest that the 16SrII group phytoplasmas found in the different symptomatic plants collected from Mailiao belong to the same strain.

### Genome assembly and comparative genomic analysis of ‘*Ca.* P. aurantifolia’ NCHU2022

**Figure 2:**
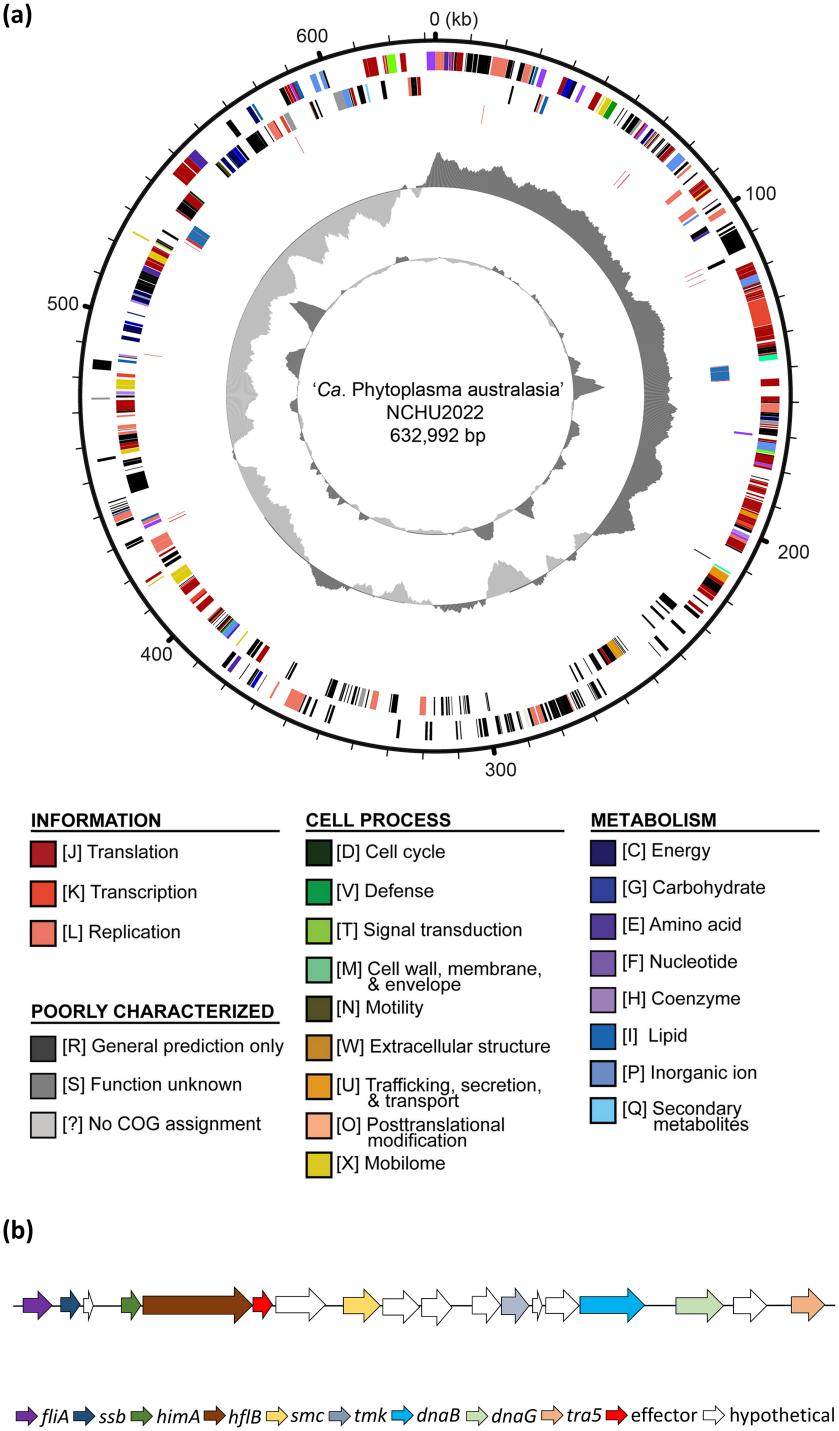
Genome architecture of ‘*Ca.* P. aurantifolia’ NCHU2022. **(a)** Circular genome map of the NCHU2022 strain is shown. Rings from the outside inward represent: (1) genome coordinates (kb). (2) predicted protein-coding genes on the forward strand, color-coded according to Clusters of Orthologous Groups (COG) functional categories; (3) predicted protein-coding genes on the reverse strand, color-coded as in (2); (4) rRNA genes (blue) and tRNA genes (red); (5) GC skew, with positive values indicated in dark gray and negative values in light gray; (6) GC content, with regions above the average shown in dark gray and below average in light gray. **(b)** Gene organization of the potential mobile unit (PMU) identified in NCHU2022, showing PMU-associated genes involved in DNA metabolism, transposition, and secretion, including f*liA, ssb, himA, hflB, smc, tmk, dnaB, dnaG*, and *tra5*. Arrows indicate gene orientation.

As a pathogen associated with the economically important crop diseases in Mailiao area, we decided to obtain the genome information of phytoplasma identified in diseased daikon. With the difficulty in establishment of an axenic culture of phytoplasma, the symptomatic daikon was used to obtain genomic DNA for preparation of genomic libraries. In order to achieve a complete phytoplasma genome, the Oxford Nanopore Technologies (ONT) sequencing and Illumina sequencing were performed to obtain long- and short-reads, respectively. For long-read sequencing, four libraries (A1–A4) with different DNA fragment sizes were constructed to generate a total of 2,853,855 reads containing 11,850,376,858 bp (Supplementary Table S5). The average read lengths ranged from 1.7 to 12.5 kb with a maximum read length of 127 kb. For short-read sequencing, one library (B1) for 150 bp paired-end sequencing was constructed to generate 57,501,728 reads containing 8,682,760,928 bp (Supplementary Table S5). Obtained reads were mapped to the reference genome of ‘*Ca.* P. aurantifolia’ NCHU2014 (16SrII group) associated with *Echinacea purpurea* witches’ broom (EpWB) disease without *de novo* assembly. During assembly processing, the potential assembly errors were manually adjusted based on the ONT long reads, and the potential sequencing errors introduced by long reads were verified using Illumina short reads. Overall, 0.68% of ONT long reads and 1.7% of Illumina short reads were mapped to the assembled genome, which consists of a circular chromosome with 632,992 bp and a plasmid with 4,225 bp (Figure 2a). The phytoplasma associated with the RsWB disease was then recognized as ‘*Ca.* P. aurantifolia’ NCHU2022 strain.

**Figure 3:**
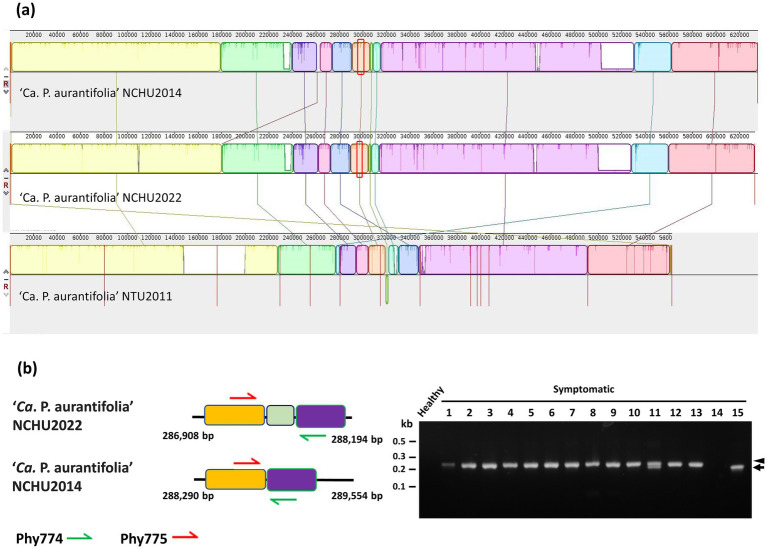
Genetic comparisons of the ‘*Ca.* P. aurantifolia’ strains found in different geographic origins. **(a)** The complete genome of ‘*Ca.* P. aurantifolia’ NCHU2022 was aligned with the complete genome of ‘*Ca.* P. aurantifolia’ NCHU2014 and the draft genome of ‘*Ca.* P. aurantifolia’ NTU2011. **(b)** Schematic diagram represented the specific primers designed for distinguishing the genome differences made by translocation between the ‘*Ca.* P. aurantifolia’ strains. PCR was conducted to examine the genome rearrangements using genomic DNA samples prepared from symptomatic daikon (*Raphanus sativus* L.) (#1), peanut (*Arachis hypogaea* L.) (#2), mungbean (*Vigna radiata L.*) (#3), soybean (*Glycine max* L.) (#4), *Ixeris chinensis* (#5), *Desmodium triflorum* (#6), *Emilia sonchifolia* (#7), *Nicotiana plumbaginifolia* Viv. (#8), *Digera muricata* L. (#9), *Parthenium hysterophorus* L. (#10), *Scaevola taccada* (#11), *Celosia argentea* L. (#12), and *Eclipta prostrata* (#13) infected by ‘*Ca.* P. aurantifolia’ strains found in Yunlin, Taiwan. Healthy daikon and the symptomatic loofah (*Luffa aegyptiaca*) (#14) infected by the 16SrVIII group ‘*Ca.* P. luffae’ NCHU2019 were used as control. The symptomatic purple coneflower (*Echinacea purpurea*) (#15) infected by the ‘*Ca.* P. aurantifolia’ NCHU2014 was found in Taichung. Arrow and arrowhead indicated the corresponding DNA fragments amplified by the specific primer sets. Non-specific signals were indicated by asterisks.

‘*Ca.* P. aurantifolia’ NCHU2022 shares 100% sequence identity in 16S rRNA, *Imp*, *tufB*, *rluA*, *degV*, *dnaD*, and TIGR00282 genes with those in ‘*Ca.* P. aurantifolia’ NCHU2014 (Supplementary Table S9). However, it is 2,592 bp shorter than that of ‘*Ca.* P. aurantifolia’ NCHU2014 (Supplementary Table S6). The comparative genomic analysis of these two closely related strains revealed that the average nucleotide identity (ANI) is 99.92% across 98.5% of their chromosomes. Consistently, pairwise genome alignment and comparison showed that their chromosomes are highly collinear except for an obvious deletion (2.4 kb) in ‘*Ca.* P. aurantifolia’ NCHU2022 (corresponding to the region from 261,100 to 263,535 bp in ‘*Ca.* P. aurantifolia’ NCHU2014) (Figure 3a). Since rearrangement remain to be observed through pairwise genome comparison, specific primers were designed to distinguish ‘*Ca.* P. aurantifolia’ NCHU2022 and ‘*Ca.* P. aurantifolia’ NCHU2014 (Figure 3b). Using specific primers Phy774/Phy775, the 206 bp DNA fragment presenting the rearrange segment of ‘*Ca.* P. aurantifolia’ NCHU2022 were amplified from the symptomatic plants (#1 to #13) collected from Mailiao, Yunlin (Figure 3b). In contrast, only 187 bp DNA fragment could be amplified from ‘*Ca.* P. aurantifolia’ NCHU2014 (#15) collected from Wufeng, Taichung (Figure 3b). As a negative control, none of them was amplified from healthy plant and the symptomatic loofah infected by ‘*Ca.* P. luffae’ NCHU2019 (#14) (Figure 3b). These results suggest that two strains (NCHU2014 and NCHU2022) of ‘*Ca.* P. aurantifolia’ share a very close genetic status and similar genomic characteristics with each other, but they carry evolutionary changes resulted from distinct environmental conditions.

### Genome annotation of ‘*Ca.* P. aurantifolia’ NCHU2022

‘*Ca.* P. aurantifolia’ NCHU2022 has a small chromosome with low GC content (24.5%), and the assembled chromosome encodes 478 protein-coding genes, 33 pseudogenes, 6 rRNA genes, and 27 tRNA genes (Supplementary Table S6). Among the protein-coding genes, there were 119 (25%) genes annotated as hypothetical proteins and only 352 (75%) genes were assigned to Clusters of Orthologous Gene (COG) categories with specific functions (Figure 2a). Further analysis by BlastKOALA for KEGG orthology assignment resulted in the assignment of 20 genes involved in the category of membrane transporter including ABC transporters (16 genes) and Sec-SRP components (4 genes), which are important for phytoplasma to import essential elements and secrete effectors (Supplementary Figures S5, S6; Supplementary Table S7).

**Figure 4:**
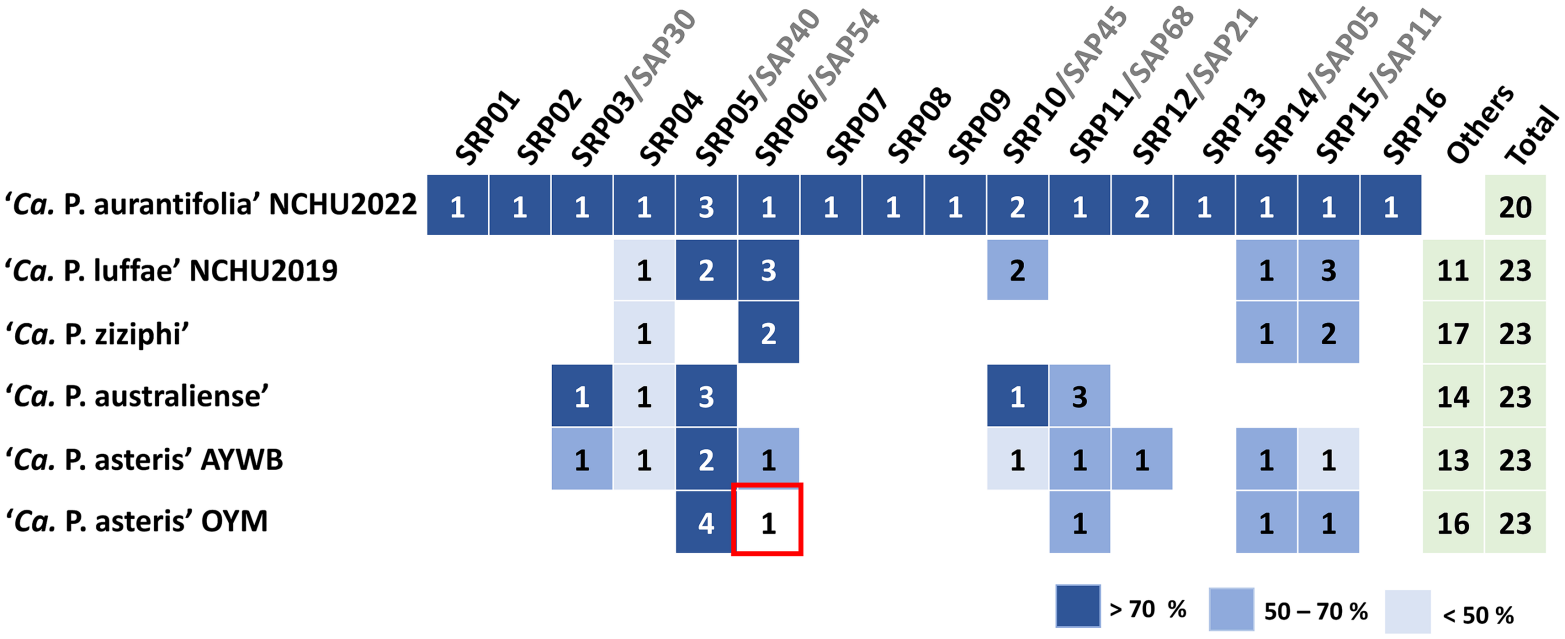
Comparative analysis of putative effectors in ‘*Ca.* P. aurantifolia’ NCHU2022 and their homologs across phytoplasma lineages. Secreted protein candidates were identified and designated as putative effectors using SignalP 6.0 and TMHMM based on the presence of N-terminal signal peptides and the absence of transmembrane domains. Comparative sequence analysis was performed by sequence similarity between secreted RsWB proteins (SRPs) and secreted AYWB proteins (SAPs) as well as others from representative phytoplasma strains, including ‘*Ca.* P. luffae’ NCHU2019, ‘*Ca. P. ziziphi*’, ‘*Ca. P. australiense*’, ‘*Ca. P. asteris*’ AYWB, and ‘*Ca. P. asteris*’ OYM, was assessed by BLASTP alignment.

In order to identify putative effectors, SignalP 6.0 was used to predict secreted proteins with a signal peptide. A total of 20 Secreted RsWB Proteins (SRPs), the potential effectors, were identified (Figure 4; Supplementary Table S8). Interestingly, 7 SRPs (SRP01, SRP02, SRP07, SRP08, SRP09, SRP13, SRP16) appeared unique in ‘*Ca.* P. aurantifolia’ NCHU2022. Among them, SRP07 was found in the Potential Mobile Unit (PMU), a putative transposable element containing *fliA* (sigma factor), *ssb* (single-stranded DNA-binding protein), *himA* (DNA-binding protein), *hflB* (ATP-dependent protease), *smc* (chromosome segregation ATPase-like protein), *tmk* (thymidylate kinase), *dnaB* (DNA helicase), *dnaG* (DNA primase), and *tra5* (transposase) (Ku et al., 2013) (Figure 2b). Only 13 SRPs displayed similarities with other secreted proteins identified in ‘*Ca.* P. luffae’ NCHU2019, ‘*Ca. P. ziziphi*’, ‘*Ca. P. australiense*’, ‘*Ca.* P. asteris’ AYWB, or ‘*Ca.* P. asteris’ OYM (Figure 4). Compare to the Secreted AYWB Proteins (SAPs) identified in ‘*Ca.* P. astetris’ AYWB, SRP05 showed >70% similarity with SAP40; SRP03, SRP06, SRP11, SRP12, and SRP14 showed 50–70% similarities with SAP30, SAP54/PHYL1, SAP68, SAP21, and SAP05, respectively; SRP10 and SRP15 showed <50% similarities with SAP45 and SAP11, respectively (Figure 4).

### Effectors responsible for the phyllody, virescence, and witches’-broom symptoms associated with RsWB disease

**Figure 5:**
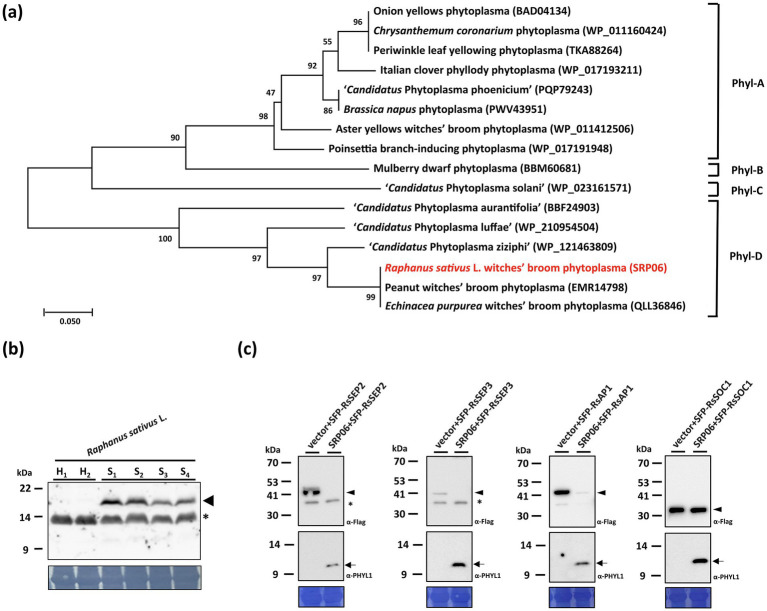
Molecular characterizations and phylogenetic analysis of the SRP06 effector of *Raphanus sativus* L. witches’-broom (RsWB) phytoplasma. **(a)** Phylogenetic tree was constructed by MEGA X software based on the phyl-A, -B, -C, and -D group of phytoplasma PHYL1/SAP54 homologs, in which the SRP06 of RsWB phytoplasma identified in this study is presented in red. **(b)** Western blotting was performed using the polyclonal antibody raised against the PHYL1/SAP54 of peanut witches’-broom phytoplasma. The specific signal of the estimated SRP06 was indicated by an arrowhead (upper panel). The non-specific signal was indicated by asterisk. The large subunit of Rubisco visualized with Coomassie Brilliant Blue staining was used as a loading control (lower panel). **(c)** Following transient co-expression in *N. benthamiana,* western blottings were performed to assess the relative abundance levels of FLAG tagged RsSEP2, RsSEP3, RsAP1 and RsSOC1 in the presence of SRP06. The specific signal of the FLAG tagged proteins and SRP06 were indicated by arrowhead (upper panel) and arrow (middle panel), respectively. The non-specific signal was indicated by asterisk. To ensure accurate loading, the large subunit of Rubisco was visualized using Coomassie Brilliant Blue staining in the lower panel.

Phylogenetic analysis revealed that SRP06 is a member of phyl-D group, which displays 51.6% sequence similarity with SAP54 of AYWB phytoplasma, a well-characterized phyl-A group effector (Figure 5a). To understand whether SRP06 is responsible for the phyllody and virescence symptoms associated with RsWB disease, the expression and biochemical activities of SRP06 were examined. Western blotting analysis revealed that SRP06 was detected only in total protein extracts from symptomatic daikon but not healthy daikon (Figure 5b). To further examine the ability of SRP06 in destabilizing *R. sativus* MTFs, SRP06 and FLAG-tagged SEPALLATA (RsSEP2 and RsSEP3), APETALA1 (RsAP1), or SUPPRESSOR OF CONSTANS OVEREXPRESSION 1 (RsSOC1) were co-expressed in *N. benthamiana* using agroinfiltration. As a result, RsSEP2, RsSEP3, and RsAP1, but not RsSOC1, were absent or greatly decreased in abundance in the presence of SRP06 compared with the vector alone (Figure 5c). These results suggest that SRP06 has the ability to degrade the *R. sativus* floral homeotic MTFs, which in turn causes the phyllody and virescence symptoms associated with RsWB disease observed in symptomatic daikon.

**Figure 6:**
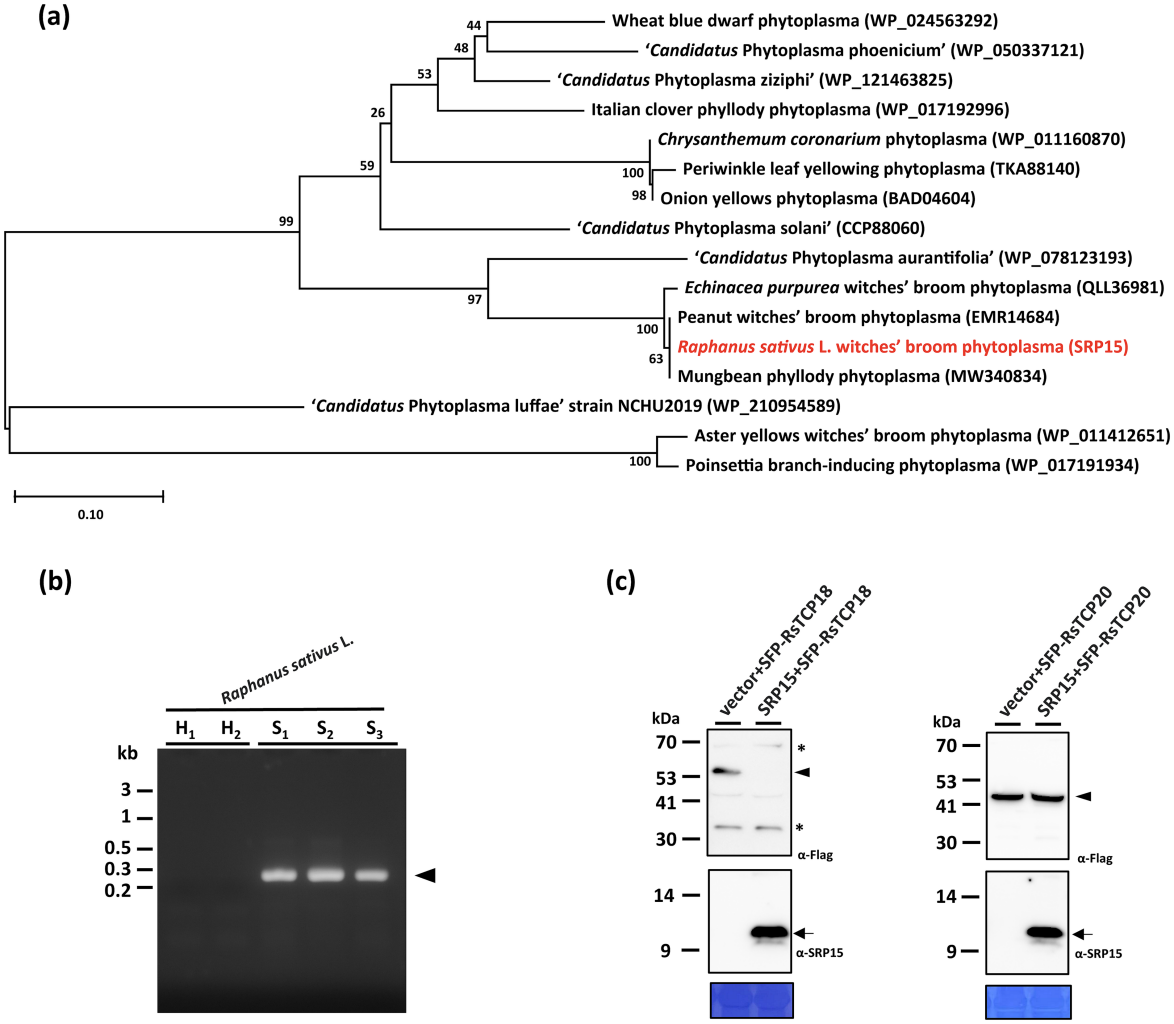
Molecular characterizations and phylogenetic analysis of the SRP15 effector of *Raphanus sativus* L. witches’-broom (RsWB) phytoplasma. **(a)** Phylogenetic tree was constructed by MEGA X software based on the phytoplasma SAP11 homologs, in which the SRP15 of RsWB phytoplasma identified in this study is presented in red. **(b)** PCR was conducted to examine the phytoplasma gene encoding SRP15 using the specific primer pair. Genomic DNA samples were prepared from healthy (H) and symptomatic (S) daikon (*R. sativus* L.). The 0.2 kb DNA fragment of *SRP15* gene was indicated by an arrowhead; **(c)** Following transient co-expression in *N. benthamiana*, western blottings were performed to assess the relative abundance levels of FLAG tagged RsTCP18 and RsTCP20 in the presence of SRP15. The specific signal of the FLAG tagged proteins and SRP15 were indicated by arrowhead (upper panel) and arrow (middle panel), respectively. The non-specific signal was indicated by asterisk. To ensure accurate loading, the large subunit of Rubisco was visualized using Coomassie Brilliant Blue staining in the lower panel.

In addition to SRP06, phylogenetic analysis revealed that SRP15 can be grouped with SAP11 homologs identified in phytoplasma strains belonging to ‘*Ca.* P. aurantifolia’ (Figure 6a). However, SRP15 only displays 37.8% sequence similarity with the well-characterized SAP11 of AYWB phytoplasma. To understand whether SRP15 is responsible for the witches’-broom symptom associated with RsWB disease, the expression and biochemical activities of SRP15 were examined. RT-PCR analysis revealed that a cDNA fragment specific for *SRP15* was amplified only in total RNA extracts from symptomatic daikon but not healthy daikon (Figure 6b). To further examine the ability of SRP15 in destabilizing *R. sativus* TCPs, SRP15 and FLAG-tagged RsTCP18 (class II CYC/TB1-TCP) or RsTCP20 (class I PCF-TCP) were co-expressed in *N. benthamiana* using agroinfiltration. As a result, RsTCP18, but not RsTCP20, was absent in abundance in the presence of SRP15 compared with the vector alone (Figure 6c). These results suggest SRP15 has the ability to degrade the *R. sativus* class II CYC/TB1-TCPs, which in turn causes the witches’-broom symptom associated with RsWB disease observed in symptomatic daikon.

### Hormonal rewiring and lignification underlie root developmental defects induced by RsWB phytoplasma

**Figure 7:**
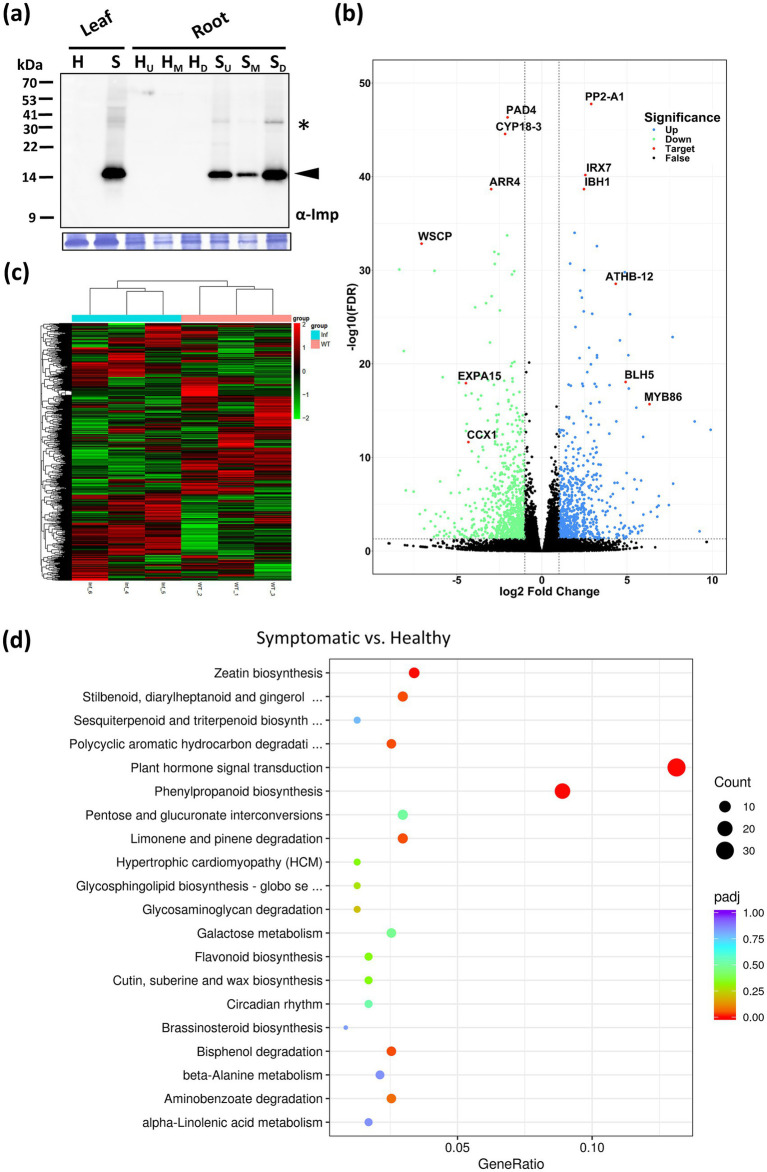
Transcriptomic analysis of daikon roots infected by RsWB phytoplasma. **(a)** Western blotting was performed using the polyclonal antibody raised against the immunodominant membrane protein (Imp) of peanut witches’-broom phytoplasma (upper panel). The specific signal of Imp (19 kDa) was indicated by an arrowhead. The large subunit of Rubisco visualized with Coomassie Brilliant Blue staining was used as a loading control (lower panel). **(b)** Volcano plot of log2 fold change of differential expression analysis for healthy and symptomatic roots of daikon. Blue and green points represent genes with gene-level fold change >2 and <−2, respectively. Black points are no significant genes. Red points represent selected genes involved in hormonal imbalance, suppression of immune regulators, induction of stress-responsive transcription factors, and reinforcement of secondary cell walls. **(c)** Hierarchical clustering heatmap of DEGs in the roots of healthy and symptomatic daikon. The color scale indicates the level of log2 fold change (blue, low; red, high). **(d)** KEGG enrichment analysis. Gene ratio and pathways are represented by the *x*-axis and *y*-axis, respectively; the size and color of the dots indicate the gene count and the level of *p* value, respectively, was performed to identify overrepresented pathways among differentially expressed genes.

As a root vegetable, daikon is highly dependent on robust underground development for yield. Infection by the RsWB phytoplasma resulted in severe root growth retardation, significantly reducing crop productivity (Figures 1a, 7a). However, this distinct phenotype, which cannot be explained by canonical SAP11 (SRP06) or SAP54 (SRP15) effectors alone, points to a broader strategy employed by RsWB to manipulate host developmental and defense programs. To investigate the molecular basis of this developmental defect, we conducted a transcriptomic analysis of healthy and symptomatic daikon roots using *de novo* assembly followed by functional annotation. Differentially expressed genes (DEGs) were defined as those with |log_2_ (fold change) | ≥ 1, and visualized in a volcano plot (Figure 7b). A total of 1,553 DEGs were identified, including 709 upregulated and 844 downregulated genes (Supplementary Tables S2, S3). Reliably, hierarchical clustering of DEGs using FPKM values consistently grouped biological replicates, demonstrating the robustness and reproducibility of the RNA-seq data (Figure 7c).

Among down-regulated genes, *PAD4* (a central regulator of SA-mediated immunity) (Feys et al., 2001), *ARR4* (a cytokinin signaling repressor) (Verma et al., 2015), *CYP18-3* (a cyclophilin-type PPIase involved in hormone response and stress) (Li et al., 2014), *expansin* (a cell wall-loosening factor) (Cosgrove, 2024), *WSCP-like* (a protease inhibitor) (Rustgi et al., 2017), and *CCX1* (a calcium exchanger essential for ion homeostasis and defense signaling) (Li et al., 2016) were notably suppressed. These changes suggest that RsWB infection impairs salicylic acid signaling, cytokinin regulation, protein folding, ion balance, and cell wall extensibility-potentially weakening host defense and promoting rigidified, stunted root growth (Figure 7b; Supplementary Table S3). Conversely, several defense- or development-associated genes were significantly up-regulated. These include *PP2-A1* (a phloem-associated lectin implicated in systemic signaling) (Beneteau et al., 2010), *IBH1-like* (a BR/GA-regulated bHLH transcription factor that represses cell elongation) (Zhiponova et al., 2014), *IRX7* (a glucuronosyltransferase involved in xylan biosynthesis and lignification) (Petersen et al., 2012), *BEL1-like* (a TALE-class homeodomain protein modulating meristem identity) (Kumar et al., 2007), *ATHB-12-like* (an ABA-inducible HD-Zip transcription factor associated with stress and growth inhibition) (Son et al., 2010), and *MYB86* (an R2R3-MYB involved in cuticle formation and abiotic stress tolerance) (Zhang et al., 2020) (Figure 7b; Supplementary Table S2). Together, these transcriptomic changes illustrate a complex host response involving hormonal imbalance, suppression of immune regulators, induction of stress-responsive transcription factors, and reinforcement of secondary cell walls. Such reprogramming is likely orchestrated by RsWB phytoplasma to promote pathogen colonization while impairing normal root architecture and storage function.

**Figure 8:**
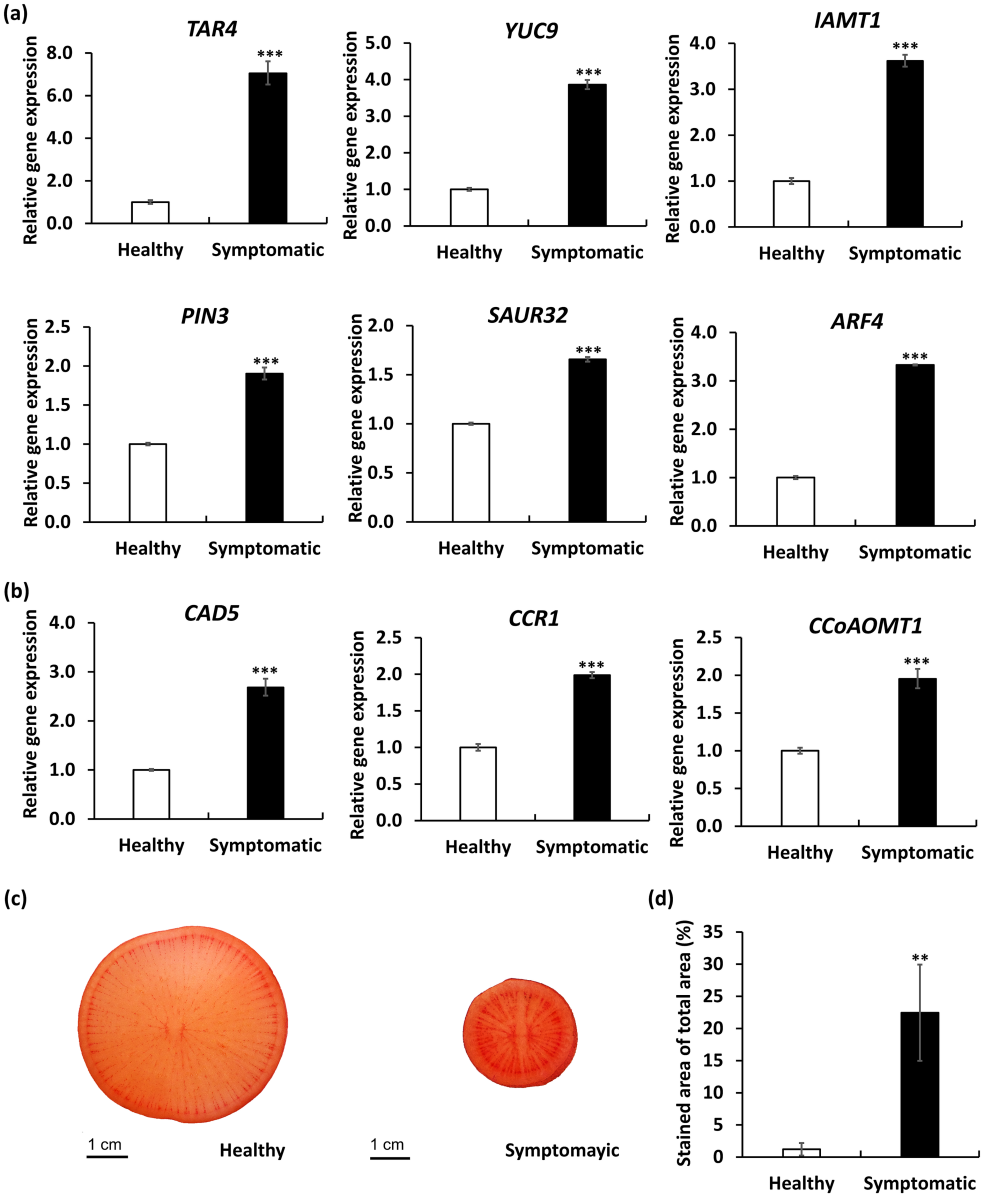
Quantitative RT-PCR (qRT-PCR) analysis and Safranin O staining of daikon roots infected by RsWB phytoplasma. **(a,b)** qRT-PCR validation of selected auxin-related genes **(a)** and lignin biosynthesis genes **(b)** exhibiting differential expression in symptomatic daikon roots. The relative expression levels of genes in healthy daikon were set to 1 after normalizing to *Actin2*. **(c)** Histological staining of symptomatic daikon roots by Safranin O. *p* values are indicated by stars, **p < 0.05, ***p < 0.001.

In order to discover the likely biological functions of the DEGs, a Kyoto Encyclopedia of Genes and Genomes (KEGG) pathway enrichment analysis was carried out. This analysis led to the mapping of DEGs onto 32 distinct KEGG pathways, with the leading 20 pathways displayed in Figure 7d. Notably, the most significantly enriched pathway is the plant hormone signal transduction pathway (ko04075, with 31 DEGs), in which, four auxin-related DEGs encoding auxin-responsive SAUR proteins and auxin response factor 4 (ARF4) were upregulated in symptomatic roots; 11 cytokinin-related DEGs encoding type-A Arabidopsis response regulators (ARR-A) were downregulated in symptomatic roots. We further validated the expression levels of key auxin-related genes including those involved in auxin biosynthesis (*TAR4*, *YUC9*) (Hofmann, 2011), auxin metabolism (*IAMT1*) (Takubo et al., 2020), auxin transport (*PIN3*) (Tao et al., 2023), and auxin responses (*SAUR32*, *ARF4*) (Cancé et al., 2022; Stortenbeker and Bemer, 2019) by qRT-PCR analysis. As a result, all auxin-related genes showed significantly elevated expression in symptomatic roots compared to healthy controls (Figure 8a). Our findings indicate that RsWB phytoplasma infection activates auxin biosynthesis and signaling pathways, impairing root development in daikon.

Such hormonal disruptions may act synergistically with ectopic lignin deposition to exacerbate root architectural defects, forming a multifaceted basis for symptom development. Indeed, phenylpropanoid biosynthesis (ko00940, with 21 DEGs), which is essential for lignin production, was identified as the second most significantly enriched KEGG pathway among the differentially expressed genes (Figure 7d). Further analysis by qRT-PCR confirmed that expression of three key lignin biosynthetic genes encoding cinnamyl alcohol dehydrogenase 5 (CAD5) (Tronchet et al., 2010), cinnamoyl-CoA reductase 1 (CCR1) (Tu et al., 2010), and caffeoyl-CoA *O*-methyltransferase 1 (CCoAOMT1) (Li et al., 1997) were significantly upregulated in symptomatic roots (Figure 8b). Consistently, safranin O staining also revealed excessive lignin accumulation in symptomatic roots (Figure 8c), in which, lignified areas were approximately five times larger than those in healthy controls (Figure 8d). These results suggest that RsWB phytoplasma infection triggers ectopic lignin deposition in daikon roots, which may represent a defensive reaction. However, the accompanying stiffening of root tissues likely impairs normal elongation and branching, thereby contributing directly to the development of root-specific disease symptoms.

## Discussion

### Phytoplasma-induced developmental reprogramming in daikon

Although phytoplasma infection in daikon has been previously reported, its molecular identity and pathogenic mechanisms remain incompletely understood, limiting our understanding of host-pathogen interactions in this economically important root crop. An early investigation by Schultz and Shaw (1991) described an outbreak in the Columbia Basin of Washington State, where symptomatic plants exhibited a premature induction of flowering, virescence, and phyllody. The disease was attributed to a beet leafhopper-transmitted virescence agent with a wide host range, tentatively classified as a mycoplasma-like organism (MLO), based on hybridization with plasmid DNA probes derived from the beet leafhopper-transmitted virescence agent (BLTVA-MLO). The “BLTVA-MLO” was assigned based on symptomatology and plasmid hybridization, however no phylogenetic resolution or genome-level characterization was done. This highlights a critical knowledge gap regarding the molecular features and host impact of daikon-infecting phytoplasmas, especially with respect to their potential manipulation of host developmental and immune processes.

In this study, we identified the causal agent of witches’-broom disease in daikon as a 16SrII-A subgroup strain of ‘*Ca.* P. aurantifolia’, designated RsWB phytoplasma, which is phylogenetically closely related to the peanut witches’-broom (PnWB) phytoplasma (Supplementary Figure S2c). Infected daikon plants exhibited phyllody, virescence, witches’-broom, and early bolting accompanied by severe root growth retardation, resembling the disease symptoms previously attributed to BLTVA-MLO (Figures 1a–e). Our genomic and functional analyses identified SAP11 and SAP54/PHYL1 homologs in RsWB phytoplasma, which are responsible for witches’-broom, phyllody, and virescence symptoms (Figures 5, 6). While effectors underlying premature bolting and root growth retardation have yet to be determined, transcriptomic analysis of symptomatic roots indicates that the root phenotype arises from coordinated transcriptional reprogramming involving hormonal imbalance, immune suppression, and secondary cell wall reinforcement (Figures 7b–d). Interestingly, this transcriptional reprogramming strongly parallels recent efforts in other root-crop systems, such as *Angelica dahurica*, where early bolting is characterized by a rapid shift from vegetative to reproductive growth, which impairs root biomass accumulation and triggers early lignification of storage tissues, ultimately reducing the yield and medicinal value of the plant (Wu et al., 2023).

In both cases, auxin-responsive genes and secondary wall regulators were strongly implicated, suggesting a conserved regulatory network under stress or abnormal developmental conditions. Notably, in RsWB-infected daikon roots, we observed upregulation of *IRX7*, a key xylan biosynthesis enzyme involved in secondary wall formation (Petersen et al., 2012), and *PP2-A1*, a phloem lectin-like protein associated with wound and defense responses (Beneteau et al., 2010)—both consistent with vascular remodeling and cell wall fortification reported in other plant pathosystems. Conversely, the downregulation of *PAD4* and *ARR4* in our study points to suppression of salicylic acid- and cytokinin-mediated defense signaling (Feys et al., 2001; Verma et al., 2015), a strategy possibly exploited by phytoplasmas to establish colonization. Interestingly, *A. dahurica* also exhibited hormone imbalance under bolting stress, with downregulation of ERFs and MYC transcription factors involved in jasmonic acid signaling, and alterations in MYB-regulated lignin biosynthesis pathways (Wu et al., 2023). This convergence supports the hypothesis that root developmental integrity under both biotic and abiotic stress relies on tightly regulated hormone crosstalk and secondary metabolism.

These findings suggests that both phytoplasma infection and premature reproductive transition can induce developmental reprogramming aimed at structural fortification rather than storage function, potentially as an adaptive or maladaptive response to stress. Such transcriptional signatures imply that lignification may be a default stress response strategy across divergent species when confronted with either internal hormonal cues or external biotic challenges. Thus, by drawing parallels between a phytoplasma-driven disease system and a hormonally deregulated bolting model, our findings suggest that phytoplasmas such as RsWB may exploit host floral transition pathways, vascular differentiation cues, and hormonal imbalances to disrupt root architecture and facilitate systemic spread.

### Host-specific modulation of auxin signaling by phytoplasmas

Auxin is a central hormone governing diverse developmental processes in plants, including cell elongation, organogenesis, vascular differentiation, and apical dominance (Vanneste et al., 2025). Disruption of auxin homeostasis or its signal transduction pathways has been consistently linked to developmental abnormalities. In phytoplasma-infected hosts, such disruptions commonly manifest as witches’-broom, phyllody, virescence, and altered flowering phenotypes, which can be traced to auxin misregulation at both transcriptional and post-transcriptional levels (Dermastia, 2019).

Mardi et al. (2015) demonstrated that infection by ‘*Ca.* P. aurantifolia’ in *Citrus aurantifolia* induces widespread transcriptomic reprogramming, prominently affecting hormone signaling networks including auxin-related pathways. Several auxin biosynthesis and response genes, including those encoding auxin response factors (ARFs) and transporters, were differentially expressed in symptomatic tissues, underscoring the centrality of auxin misregulation in symptom development. Notably, the expression patterns varied across organs and disease stages, pointing to a host- and tissue-specific modulation of auxin dynamics. A similar phenomenon was observed in *Ziziphus jujuba* infected by ‘*Ca. P. ziziphi*’, where floral tissues exhibited heightened sensitivity to infection-induced transcriptional reprogramming, particularly within the auxin signaling pathway—consistent with the observed floral malformations and phyllody symptoms (Ma et al., 2020).

Beyond transcriptional reprogramming, phytoplasma infection also perturbs auxin signaling at the post-transcriptional level through microRNA (miRNA) regulation. In *Citrus aurantifolia*, infection by ‘*Ca.* P. aurantifolia’ led to differential expression of several miRNAs, including miR160, miR166, and miR167, which target key components of the auxin signaling cascade such as ARFs and the auxin receptor TIR1 (Ehya et al., 2013). This miRNA-mediated suppression of auxin perception and response suggests a strategic modulation of hormone pathways by the pathogen. Interestingly, while miR393—known to repress TIR1 and enhance immunity in Arabidopsis under flg22-triggered responses (Staiger et al., 2013)—was implicated in this context, infected lime trees still exhibited elevated IAA levels (Ehya et al., 2013). This paradox implies that phytoplasmas may hijack or override typical miRNA feedback mechanisms, leading to auxin overaccumulation despite regulatory repression.

Together, these findings underscore the complexity and host-specificity of auxin signaling responses during phytoplasma infection. Phytoplasmas appear to deploy multifaceted strategies—spanning transcriptional reprogramming, miRNA-mediated regulation, and hormonal feedback disruption—to tailor developmental outcomes in a tissue-dependent manner, facilitating systemic colonization and symptom expression.

### Root-specific auxin disruption in RsWB-infected daikon

While shoot proliferation and floral organ abnormalities are widely recognized as common indicators of phytoplasma infections across many plant species (Kumari et al., 2019; Namba, 2019), the effects on underground tissues, particularly root development, have been less extensively studied. For early root development, auxin is critical for stimulating meristematic activity and initiating lateral roots (Roychoudhry and Kepinski, 2022). During initial root thickening stages, elevated auxin levels promote cambial cell proliferation and vascular differentiation. However, transitioning effectively into the storage phase necessitates a decrease in auxin concentration, permitting cell enlargement, starch accumulation, and tissue maturation (Kondhare et al., 2021). Thus, fine-tuned auxin homeostasis is indispensable for proper root morphogenesis. Disturbances in this delicate hormonal balance, whether induced externally or via pathogen interaction, can drastically impact root structure and overall yield.

In the case of RsWB-infected daikon, a prominent symptom is severe root growth retardation accompanied by early bolting and premature reproductive development (Figures 1a,b). Transcriptome profiling of symptomatic roots revealed altered expression of auxin-related genes, including key regulators such as *ARR4* and *CYP18-3*, suggesting a shift toward auxin dominance at the expense of coordinated cytokinin signaling regulation (Figure 7b). To validate the transcriptomic observations, we performed qRT-PCR to quantify the expression levels of key auxin-related genes spanning various functional domains: biosynthesis (*TAR4*, *YUC9*), metabolism (*IAMT1*), transport (*PIN3*), and signal response (*SAUR32*, *ARF4*). All examined genes exhibited significantly elevated expression in symptomatic roots relative to healthy controls, providing strong molecular support that RsWB infection promotes auxin biosynthesis and signaling activation (Figure 8a).

Together, these findings reveal a distinct host-pathogen interaction in which the phytoplasma compromises daikon’s root developmental program through multilayered interference with auxin dynamics. Unlike many other phytoplasma-infected species where aboveground structures are the primary targets, daikon displays a belowground-centered pathology that underscores the importance of root-specific hormone crosstalk and the need to further investigate effector functions that may act within the root niche.

### Genomic Insights into Effector-Mediated Pathogenicity of Broad-Host-Range 16SrII-A ‘*Ca. P. aurantifolia*’

Phytoplasma genomes are characteristically small, highly AT-rich, and streamlined, typically ranging from 576 to 960 kb (Wei and Zhao, 2022). A major contributor to their genomic plasticity is the presence of potential mobile units (PMUs), which are clusters of mobile genetic elements that mediate horizontal gene transfer and genomic rearrangements (Bai et al., 2006). Variation in PMU abundance is closely linked to genome size differences among phytoplasmas, as strains with multiple PMUs often have larger genomes and a greater capacity for effector diversification. In this study, the genome of the ‘*Ca. P. aurantifolia*’ strain NCHU2022 was determined to be 633 kb, placing it at the lower end of the known phytoplasma genome size range, and it contains only a single PMU. By contrast, the genome of ‘*Ca. P. luffae*’ is 769 kb and contains 13 PMUs (Huang et al., 2022). The low PMU copy number in NCHU2022 likely reflects cumulative historical recombination and rearrangement events, producing a streamlined genome that is well adapted to diverse environmental conditions and host species. Despite its reduced mobile genetic content, the genome retains essential virulence determinants, including key effectors SRP06 and SRP15, which are critical for pathogenicity. Transient expression assays in *Nicotiana benthamiana* demonstrated that SRP06, a homolog of SAP54, induces phyllody by promoting the degradation of RsSEP2, RsSEP3, and RsAP1 (Figure 5c), whereas SRP15, a homolog of SAP11, triggers witches’-broom by destabilizing class II CYC/TB1-TCPs (Figure 6c). Beyond their developmental effects, these effectors also have been demonstrated to play important roles in disease dissemination by influencing insect vector interactions. For example, by targeting floral regulators such as MADS-box transcription factors, SAP54 increases floral abnormalities that enhance leafhopper attraction, facilitating mating and subsequent phytoplasma acquisition and transmission (Orlovskis et al., 2025). Similarly, by destabilizing CIN-TCPs, SAP11 suppresses *LOX2* expression and jasmonic acid biosynthesis, weakening plant defenses and enhancing the fecundity of the leafhopper *Macrosteles quadrilineatus* (Sugio et al., 2011a). Together, these effectors modify host growth and physiology in ways that favor insect vectors, ultimately promoting rapid disease spread and persistence in agricultural ecosystems. In addition to vector-mediated transmission, the broad host range of the 16SrII-A subgroup ‘*Ca. P. aurantifolia*’ likely contributes to local disease spread by providing natural reservoir hosts. Field surveys in Yunlin revealed that this phytoplasma infects not only daikon but also a wide array of crops and weeds, including *Ixeris chinensis, Desmodium triflorum, Emilia sonchifolia, Nicotiana plumbaginifolia Viv., Digera muricata L., Parthenium hysterophorus L., Scaevola taccada, Celosia argentea L.,* and *Eclipta prostrata* (Figure S4, Table S4), with all symptomatic plants exhibiting prominent witches’-broom and phyllody phenotypes (Chen et al., 2021; Chien et al., 2020a,b,c; Liao et al., 2022; Liu et al., 2015; Mejia et al., 2022; Wang et al., 2021). Although the precise mechanisms underlying this broad host range remain unclear, the combined effects of SAP11 and SAP54 on host development and insect vector interactions likely play central roles in facilitating phytoplasma transmission across diverse plant species.

## Data Availability

The complete genome sequence of ‘Ca. Phytoplasma aurantifolia’ strain NCHU2022 has been deposited in the NCBI database under GenBank accession numbers CP097312 (chromosome) and CP097313 (plasmid). The genome sequencing project, including the associated raw sequencing reads, is available under BioProject accession number PRJNA834592.

## Footnotes

^1^ https://plantpathology.ba.ars.usda.gov/cgi-bin/resource/iphyclassifier.cgi
