# Supplementary material for: Comprehensive genomic and functional characterization of a phytoplasma associated with root retardation, early bolting, witches’-broom, and phyllody in daikon (Raphanus sativus L.)
Source: Front Microbiol. 2025 Sep 9;16:1654928. doi: 10.3389/fmicb.2025.1654928 (PMC12454383; doi:10.3389/fmicb.2025.1654928)
Supplement: Supplementary file 1 [file Data_Sheet_1.docx]

**Supporting information legends**


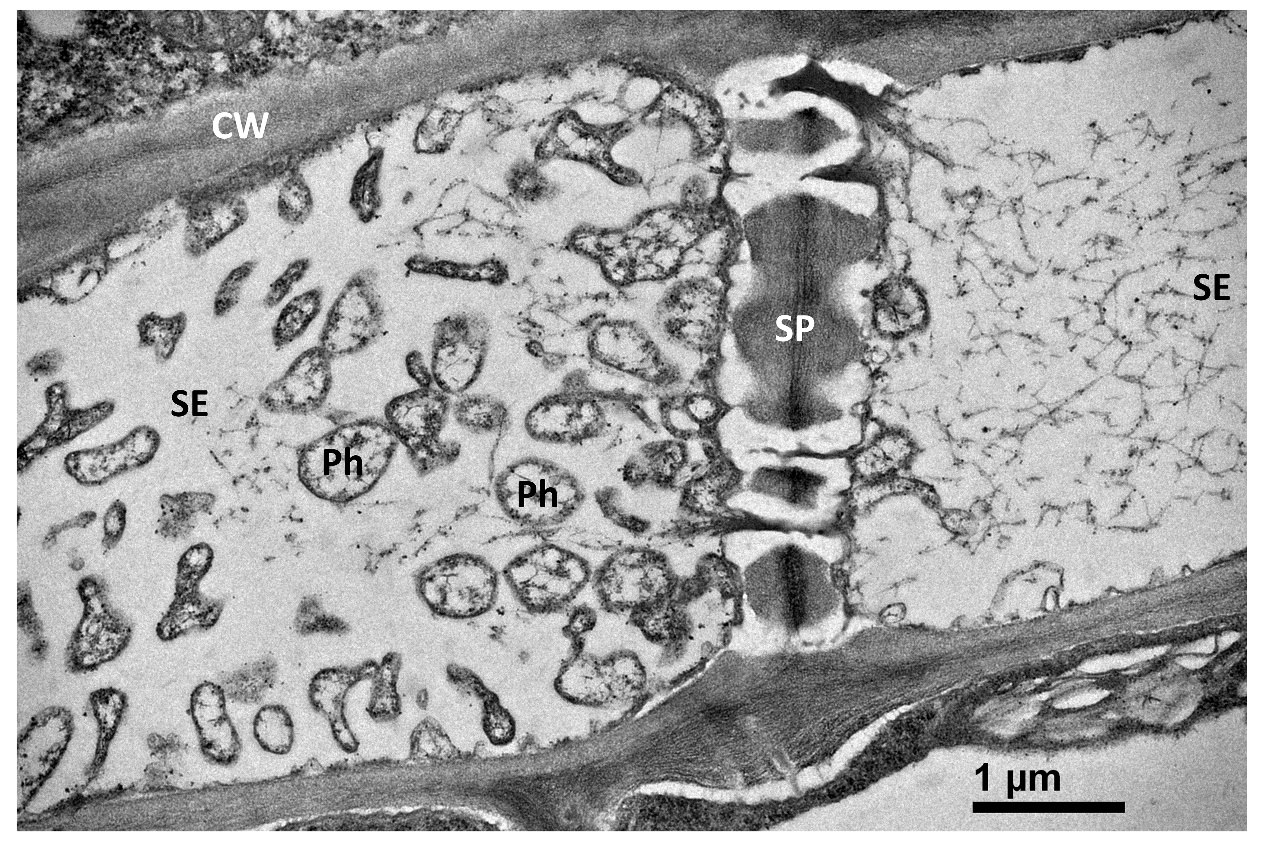


**Figure S1.** Transmission electron micrograph of the phytoplasma associated with symptomatic daikon. Pleomorphic phytoplasma cells inhabit the sieve elements of leaves collected from the symptomatic daikon. CW, cell wall; Ph, phytoplasma; SE, sieve element; SP, sieve plate.


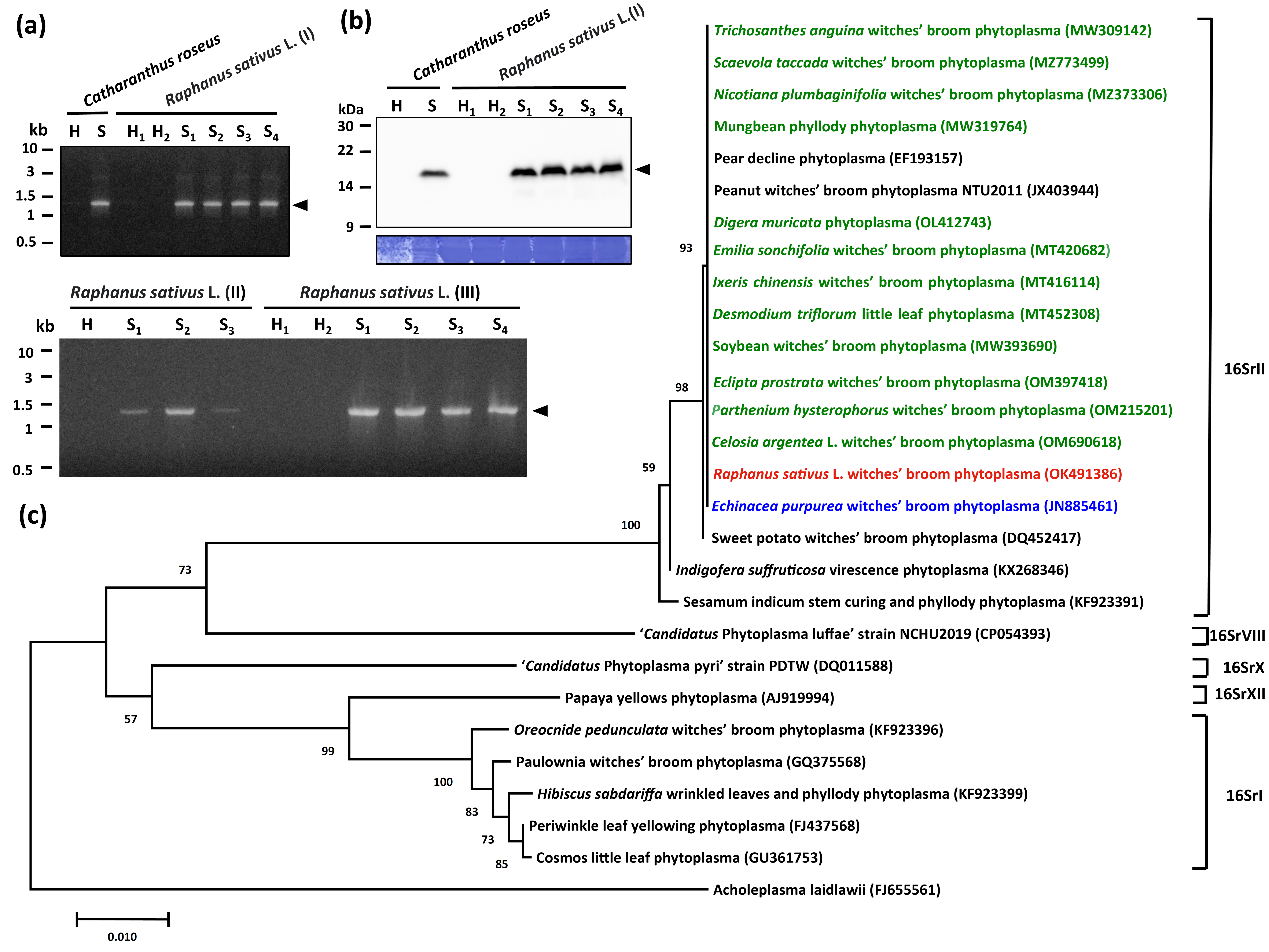


**Figure S2.** Molecular characterizations and phylogenetic analysis of *Raphanus sativus* L. witches’ broom phytoplasma. (a) Nested PCR was employed to examine the phytoplasma 16S rRNA gene using genomic DNA samples prepared from healthy (H) and symptomatic (S) daikon (*R. sativus* L.) collected in 23°45'13.0"N, 120°15'28.2"E (I), 23°45'56.2"N, 120°14'49.4"E (II), and 23°45'44.7"N, 120°15'00.1"E (III). *Catharanthus roseus* infected by the 16SrII-A subgroup *Echinacea purpurea* witches'- broom phytoplasma was used as a positive control. The 1.2 kb DNA fragment of the 16S rRNA gene was indicated by arrowhead. (b) Western blotting was performed using the polyclonal antibody raised against the immunodominant membrane protein (Imp) of peanut witches’- broom phytoplasma (upper panel). The specific signal of Imp (19 kDa) was indicated by an arrowhead. The large subunit of Rubisco visualized with Coomassie Brilliant Blue staining was used as a loading control (lower panel). (c) Phylogenetic tree was constructed based on the R16F2n/R16R2 fragment of the 16S rRNA gene identified from 27 phytoplasma strains in Taiwan, in which the RsWB phytoplasma identified in this study is presented in red. *Acholeplasma laidlawii* served as an outgroup.


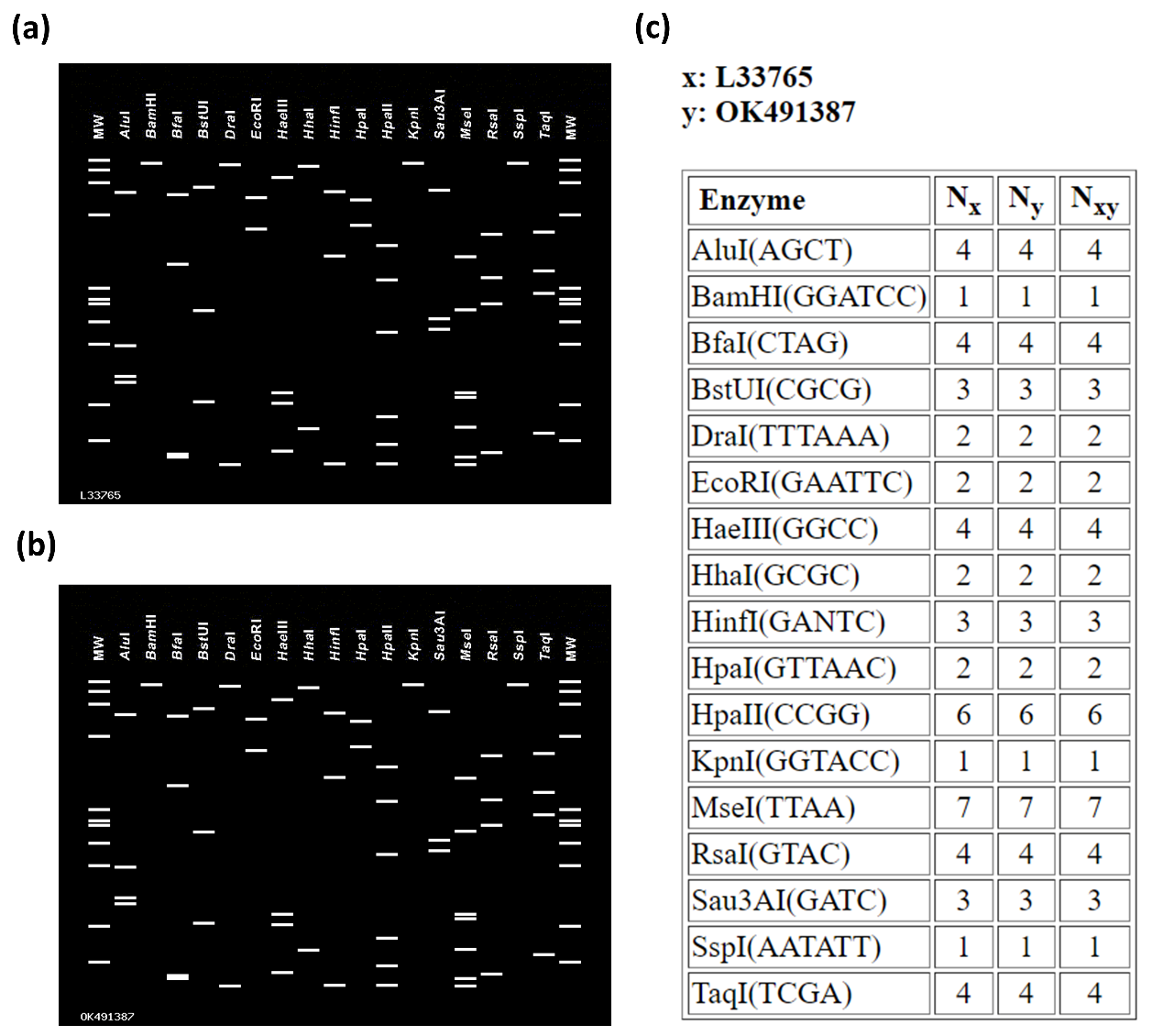


**Figure S3.** Virtual RFLP patterns derived from *i*PhyClassifier. Virtual RFLP patterns were generated by *in silico* digestion of the 1.2 kb DNA fragment of the 16S rRNA gene identified from *Raphanus sativus* L. witches’ broom phytoplasma (accession No. OK491387) (a) and peanut witches’ broom phytoplasma (accession No. L33675) (b). The comparison details of virtual RFLP patterns of MZ373306 (x) and L33675 (y) are presented (C). Nx and Ny are the total number of DNA fragments resulting from enzyme digestions in x and y, respectively. Nxy is the number of DNA fragments shared by x and y.


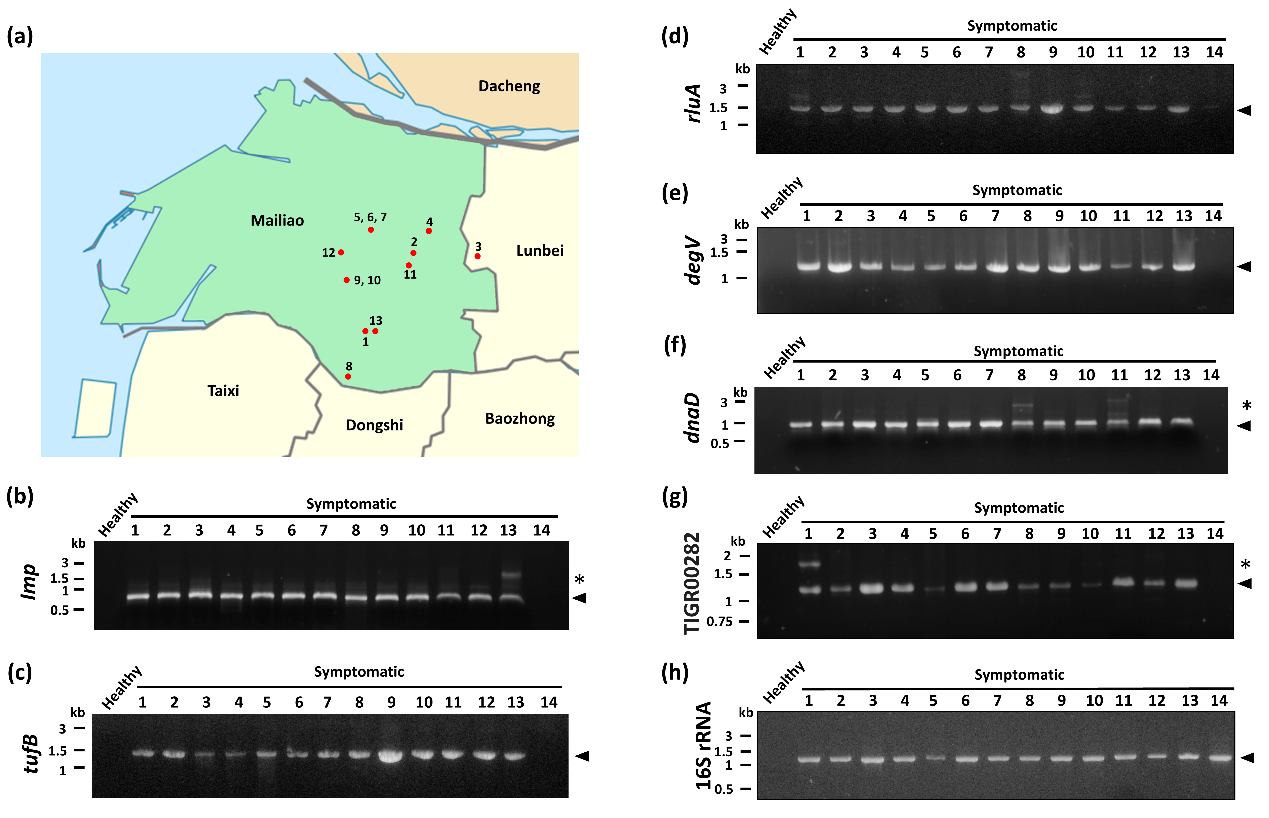


**Figure S4.** Examinations of genetic correlation of the ‘*Ca.* P. aurantifolia’ strains found in Yulin, Taiwan. Map (a) and PCR examinations (b-h) of the ‘*Ca.* P. aurantifolia’ strains found in Yunlin, Taiwan. PCR was conducted to examine the *Imp* (b), *tufB* (c), *rluA* (d), *degV* (e), *dnaD* (f)*,* TIGR00282 (g), and 16S rRNA (h) genes using genomic DNA samples prepared from symptomatic daikon (*Raphanus sativus* L.) (#1), peanut (*Arachis hypogaea* L.) (#2), mungbean (*Vigna radiata L.*) (#3), soybean (*Glycine max* L.) (#4), *Ixeris chinensis* (#5), *Desmodium triflorum* (#6), *Emilia sonchifolia* (#7), *Nicotiana plumbaginifolia* Viv. (#8), *Digera muricata* L. (#9), *Parthenium hysterophorus* L. (#10), *Scaevola taccada* (#11), *Celosia argentea* L. (#12), and *Eclipta prostrata* (#13) infected by ‘*Ca.* P. aurantifolia’. Healthy daikon and the symptomatic loofah (*Luffa aegyptiaca*) (#14) infected by the 16SrVIII group ‘*Ca.* P. luffae’ NCHU2019 were used as control. Arrowheads indicated the corresponding DNA fragments of the *Imp*, *tufB*, *rluA*, *degV*, *dnaD*, TIGR00282, and 16S rRNA genes amplified by the specific primer sets. Non-specific signals were indicated by asterisks.


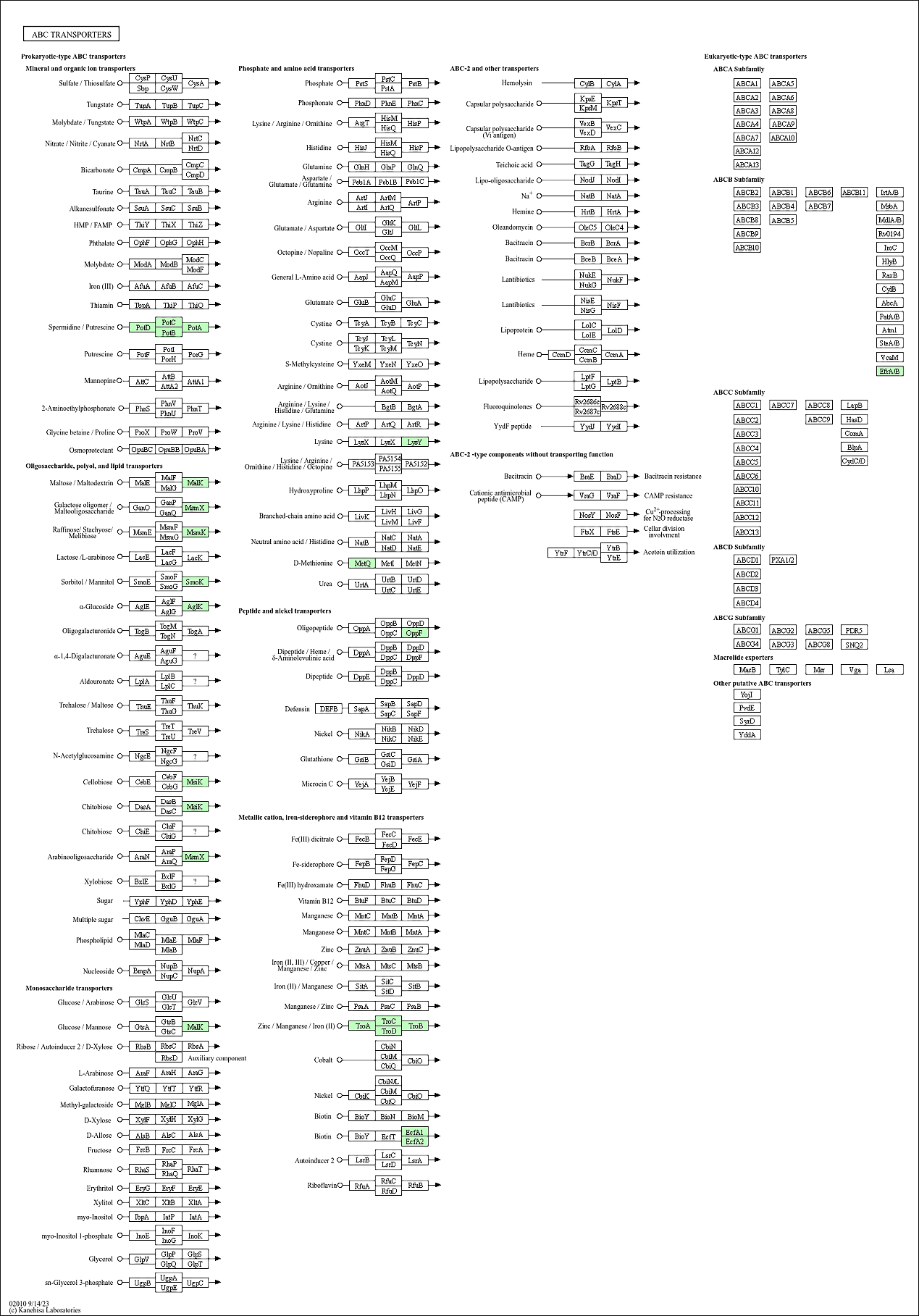


**Figure S5.** Prediction of ABC transporters in ‘*Ca. P.* aurantifolia’ strain NCHU2022. ABC (ATP-binding cassette) transporters in the phytoplasma genome were classified into prokaryote-type importers, ABCC and ABCD exporters, eukaryote-type ABC transporters, and other ABC-associated proteins. Green-highlighted genes indicate those identified in the genome.


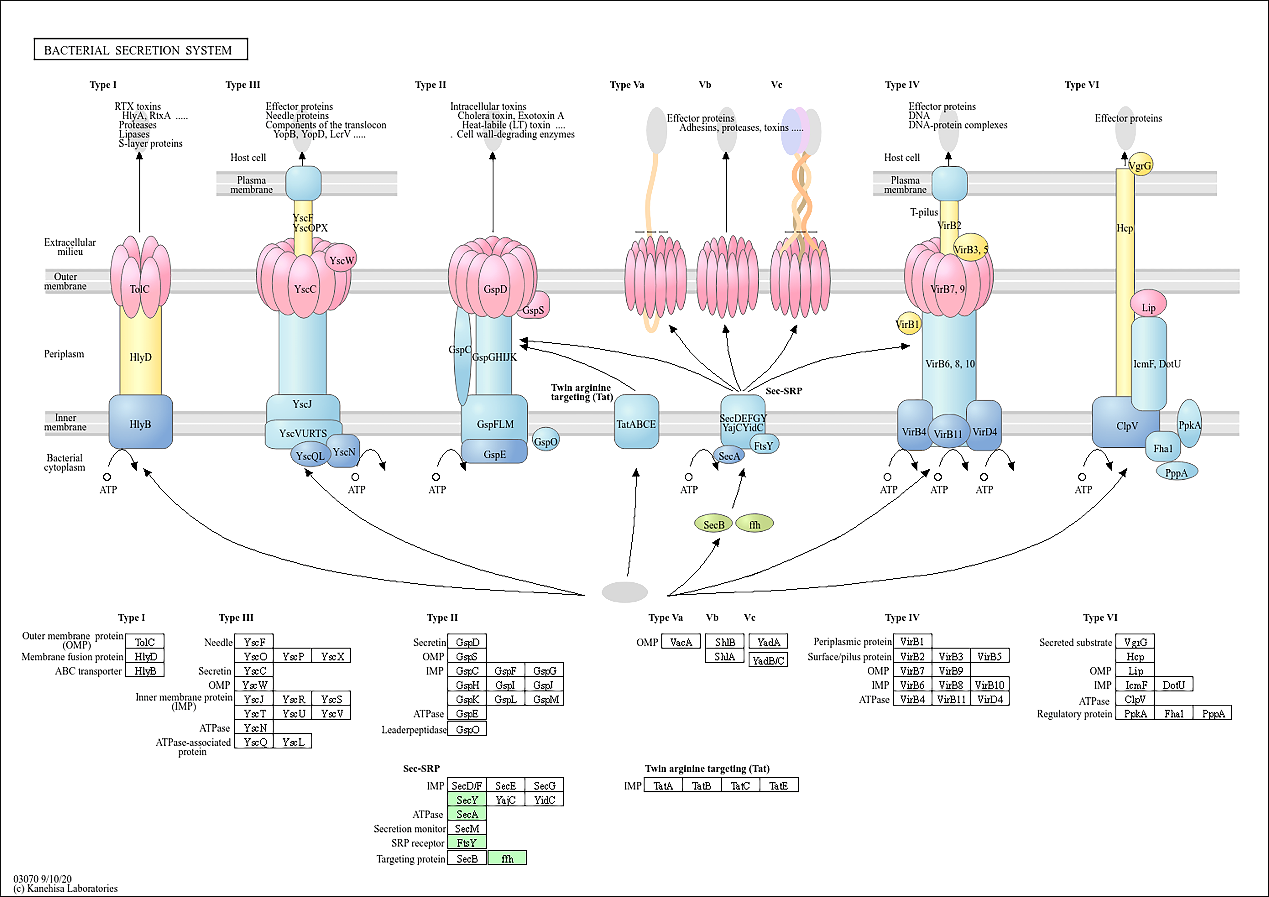
**Figure S6.** The types of bacterial secretion systems. Six distinct secretion systems have been shown to mediate protein export through the inner and outer membranes of Gram-negative bacteria. Two secretion systems, including the Sec pathway and the twin-arginine (Tat) pathway, commonly translocate proteins across the single membrane in Gram-positive bacteria. The green boxes indicate the preprotein translocase subunits, which are excited in *Raphanus sativus* L. witches’ broom phytoplasma.
