# Supplementary figures and images for: Comprehensive genomic and functional characterization of a phytoplasma associated with root retardation, early bolting, witches’-broom, and phyllody in daikon (Raphanus sativus L.)
Source: Front Microbiol. 2025 Sep 9;16:1654928. doi: 10.3389/fmicb.2025.1654928 (PMC12454383; doi:10.3389/fmicb.2025.1654928)

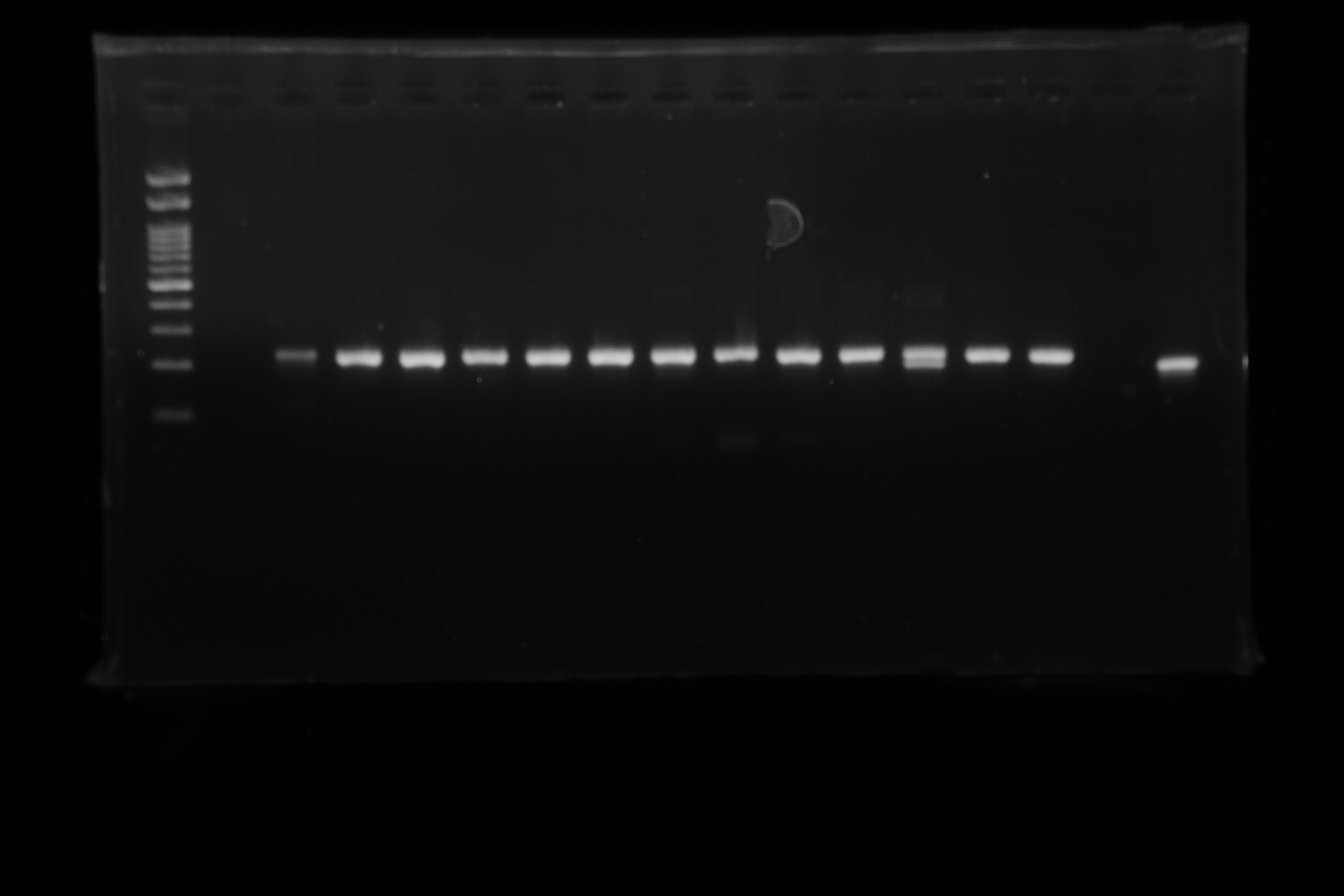

Supplement: Supplementary file 2 [file Data_Sheet_2.zip › F3.tif]

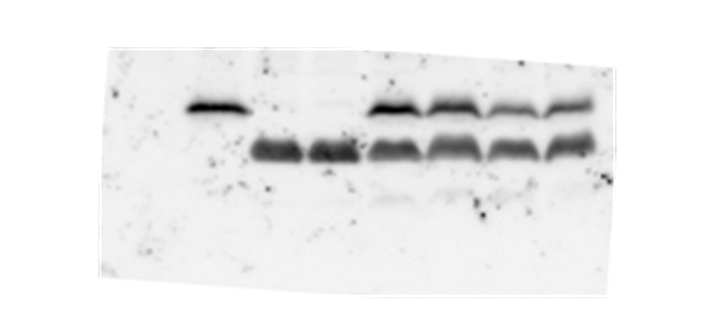

Supplement: Supplementary file 2 [file Data_Sheet_2.zip › F5b-1.tif]

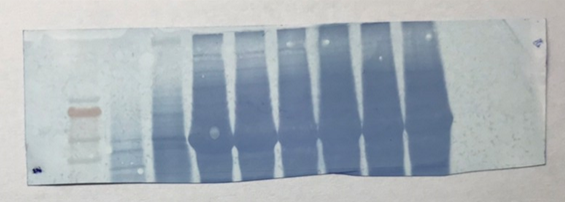

Supplement: Supplementary file 2 [file Data_Sheet_2.zip › F5b-2.tif]

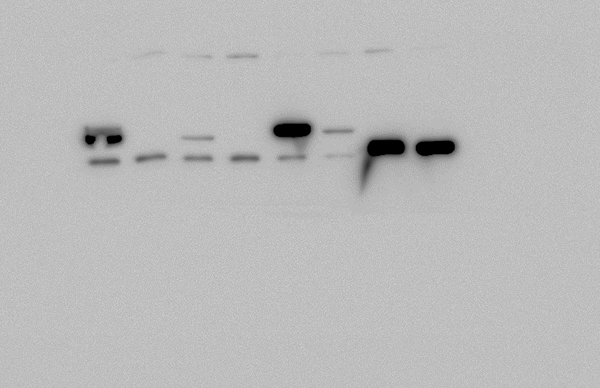

Supplement: Supplementary file 2 [file Data_Sheet_2.zip › F5c-1.tif]

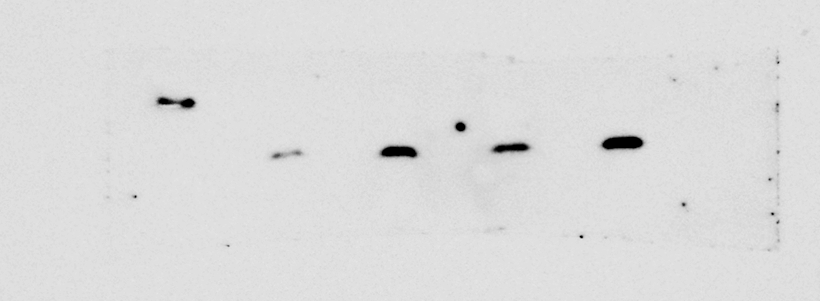

Supplement: Supplementary file 2 [file Data_Sheet_2.zip › F5c-2.tif]

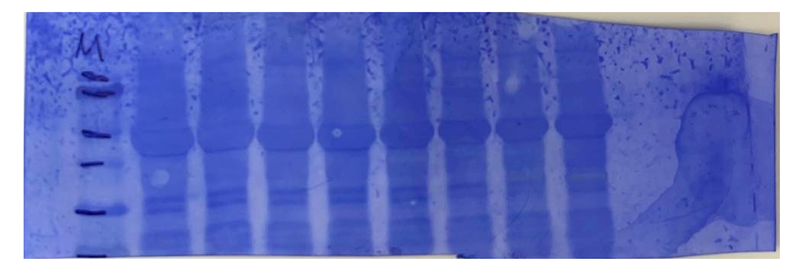

Supplement: Supplementary file 2 [file Data_Sheet_2.zip › F5c-3.tif]

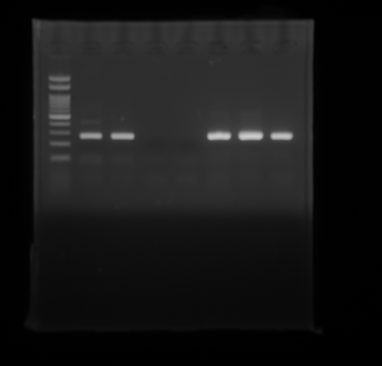

Supplement: Supplementary file 2 [file Data_Sheet_2.zip › F6b.tif]

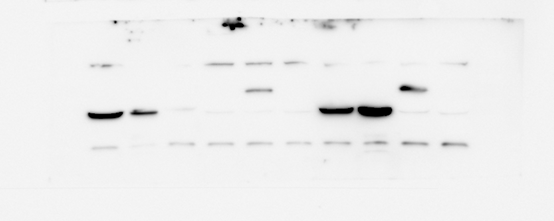

Supplement: Supplementary file 2 [file Data_Sheet_2.zip › F6c-1.tif]

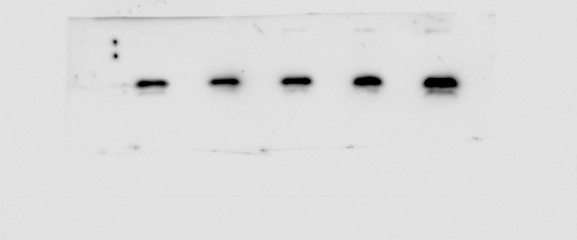

Supplement: Supplementary file 2 [file Data_Sheet_2.zip › F6c-2.tif]

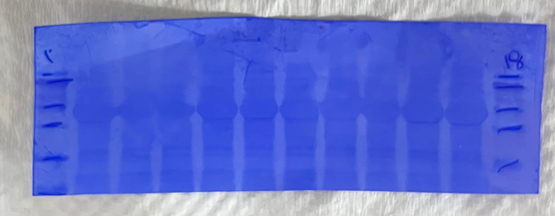

Supplement: Supplementary file 2 [file Data_Sheet_2.zip › F6c-3.tif]

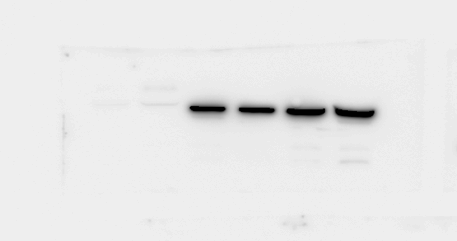

Supplement: Supplementary file 2 [file Data_Sheet_2.zip › F6c-4.tif]

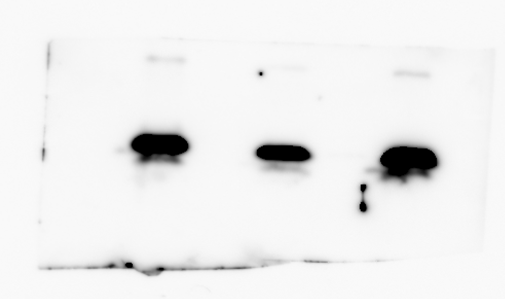

Supplement: Supplementary file 2 [file Data_Sheet_2.zip › F6c-5.tif]

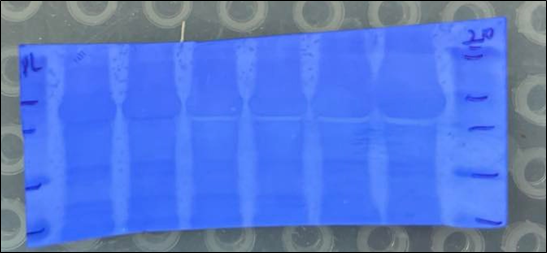

Supplement: Supplementary file 2 [file Data_Sheet_2.zip › F6c-6.tif]

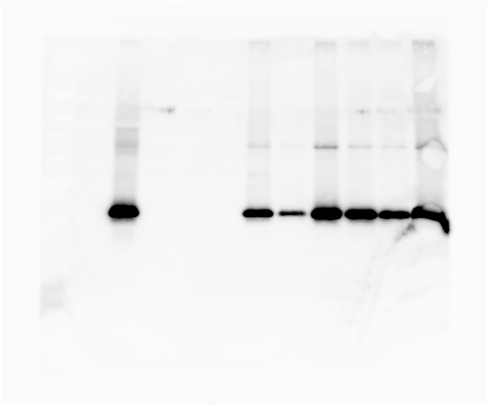

Supplement: Supplementary file 2 [file Data_Sheet_2.zip › F7a-1.tif]

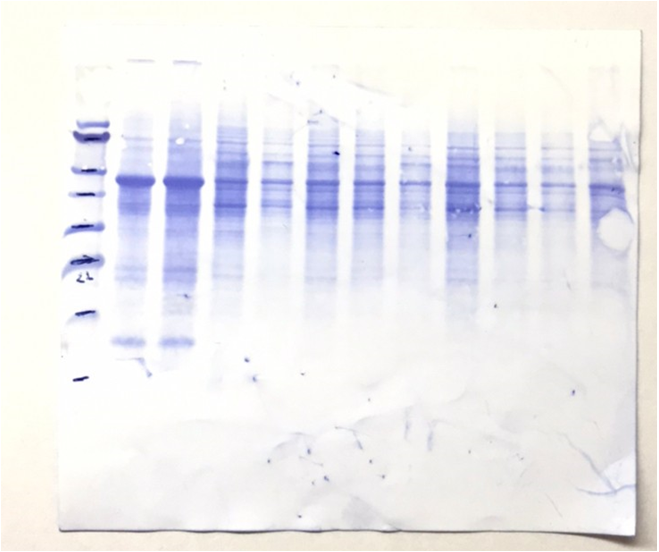

Supplement: Supplementary file 2 [file Data_Sheet_2.zip › F7a-2.tif]

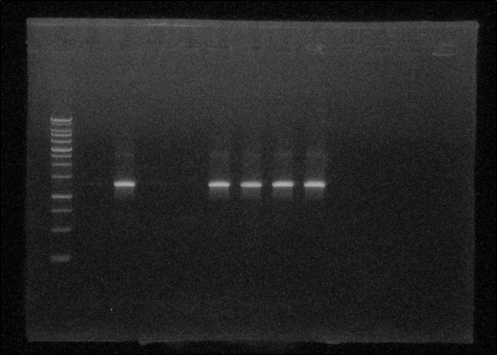

Supplement: Supplementary file 2 [file Data_Sheet_2.zip › S2a-1.tif]

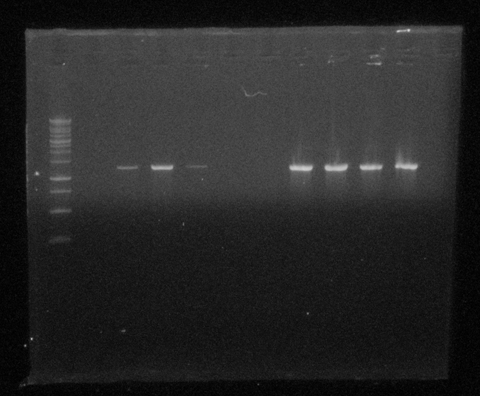

Supplement: Supplementary file 2 [file Data_Sheet_2.zip › S2a-2.tif]

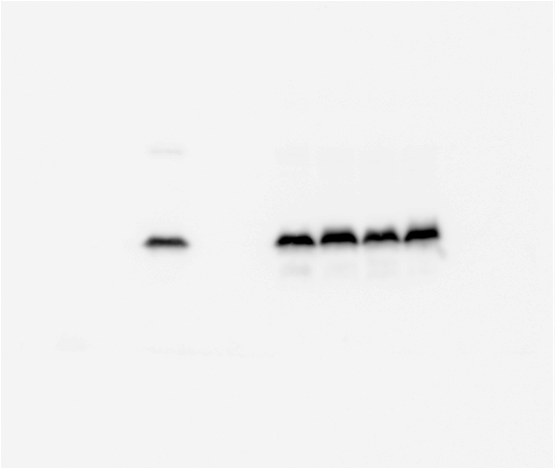

Supplement: Supplementary file 2 [file Data_Sheet_2.zip › S2b-1.tif]

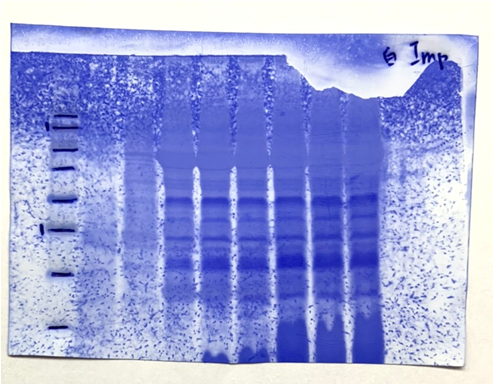

Supplement: Supplementary file 2 [file Data_Sheet_2.zip › S2b-2.tif]

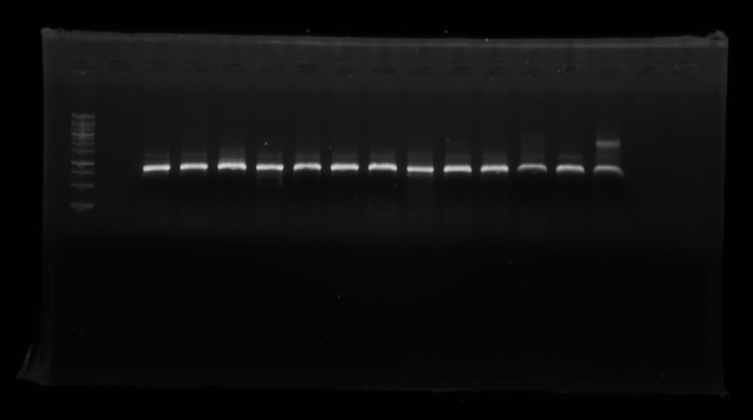

Supplement: Supplementary file 2 [file Data_Sheet_2.zip › S4b.tif]

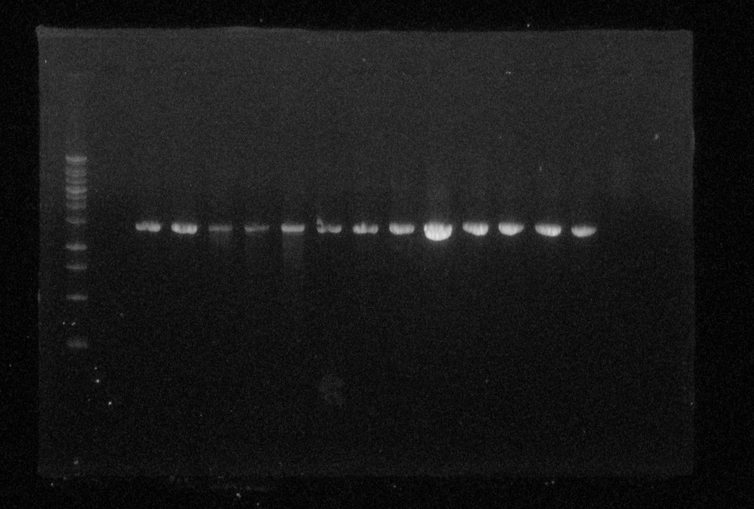

Supplement: Supplementary file 2 [file Data_Sheet_2.zip › S4c.tif]

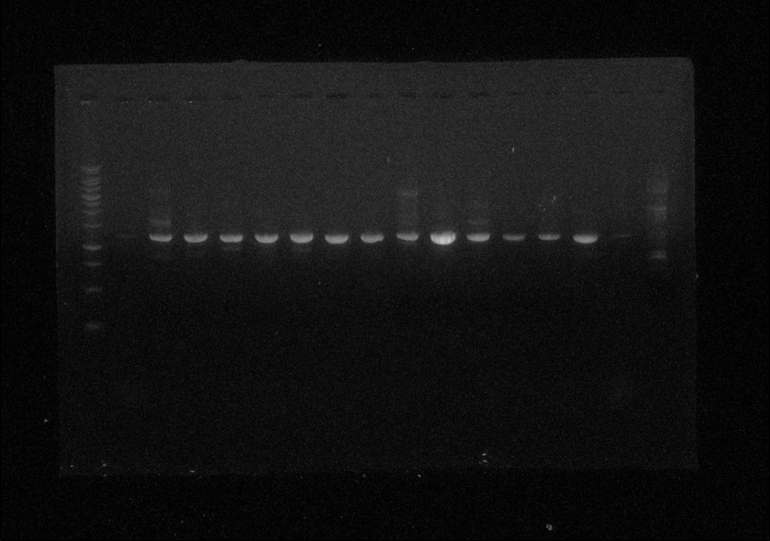

Supplement: Supplementary file 2 [file Data_Sheet_2.zip › S4d.tif]

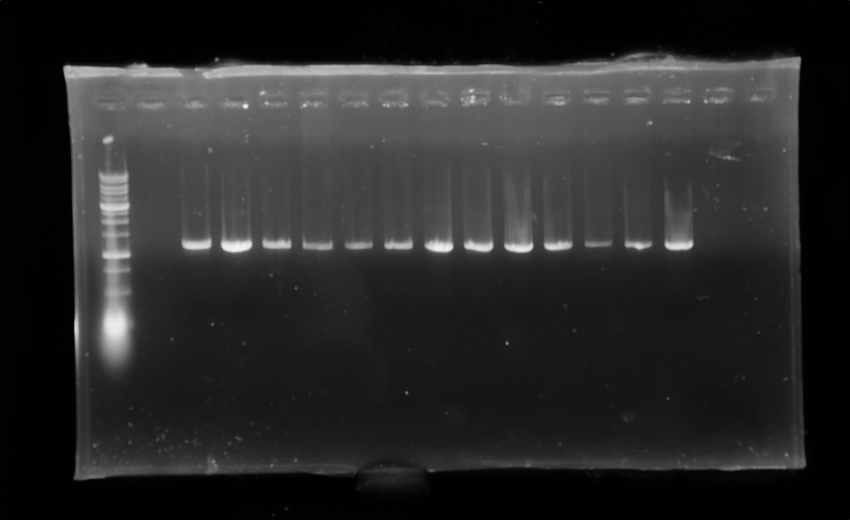

Supplement: Supplementary file 2 [file Data_Sheet_2.zip › S4e.tif]

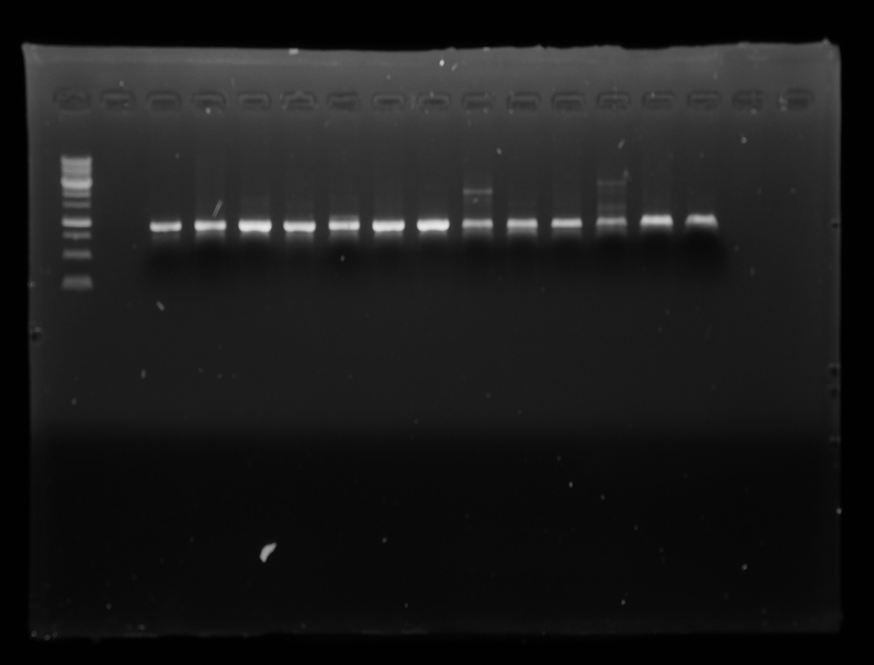

Supplement: Supplementary file 2 [file Data_Sheet_2.zip › S4f.tif]

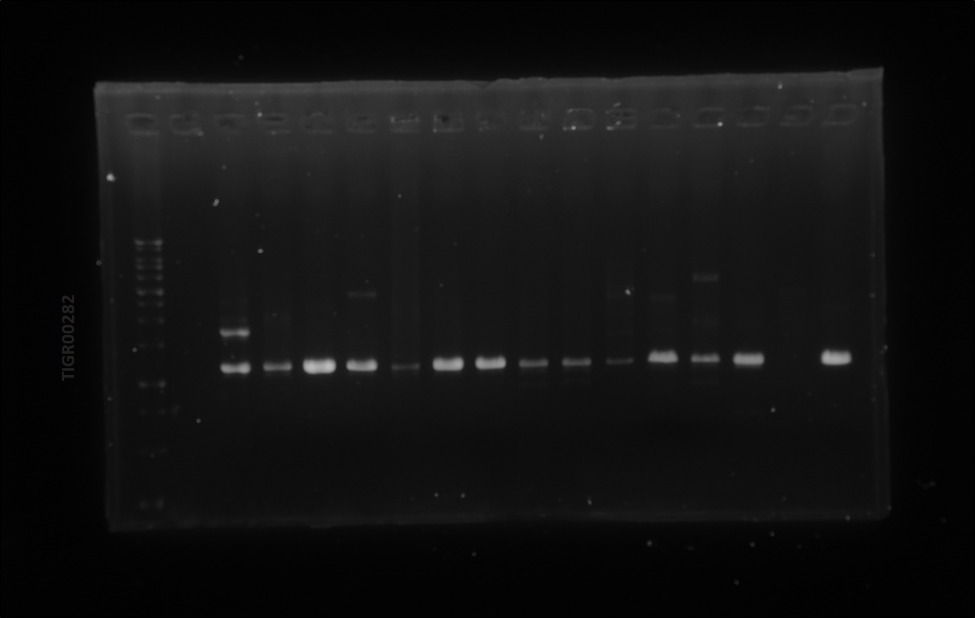

Supplement: Supplementary file 2 [file Data_Sheet_2.zip › S4g.tif]

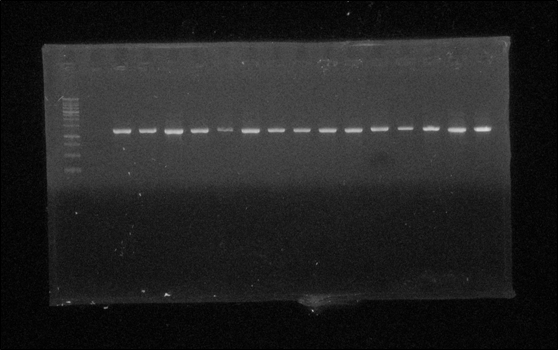

Supplement: Supplementary file 2 [file Data_Sheet_2.zip › S4h.tif]
